# Impact of Prenatal Dietary Soy on Cerebellar Neurodevelopment and Function in Experimental Fetal Alcohol Spectrum Disorder

**DOI:** 10.3390/nu17050812

**Published:** 2025-02-26

**Authors:** Suzanne M. de la Monte, Ming Tong, Jason Ziplow, Princess Mark, Stephanie Van, Van Ahn Nguyen

**Affiliations:** 1 Departments of Pathology and Laboratory Medicine, Neurosurgery, and Neurology, Rhode Island Hospital, Providence, RI 02903, USA; 2 Women & Infants Hospital, Brown University Health, Providence, RI 02905, USA; 3 Alpert Medical School of Brown University, Providence, RI 02903, USA; 4 Department of Medicine, Rhode Island Hospital, Brown University Health, Providence, RI 02903, USA; 5 Departments of Neuroscience and Biology, Brown University, Providence, RI 02903, USA; ziplowj@chop.org (J.Z.); svan1@jhu.edu (S.V.);

**Keywords:** fetal alcohol spectrum disorder, cerebellum, dietary soy, insulin signaling, Notch, behavior, rat model, Wnt, gene expression, prenatal alcohol exposure

## Abstract

**Background:** Prenatal alcohol exposure (PAE) models can cause neurodevelopmental abnormalities like those observed in fetal alcohol spectrum disorder (FASD). Previous studies link experimental PAE effects in the brain to impaired signaling through insulin/IGF and Notch pathways that mediate neuronal survival, growth, migration, energy metabolism, and plasticity. Importantly, concurrent administration of peroxisome proliferator-activated receptor agonists or dietary soy prevented many aspects of FASD due to their insulin-sensitizing, anti-inflammatory, and antioxidant properties. **Objective**: To determine if dietary soy interventions during pregnancy would be sufficient to normalize central nervous system structure and function, we examined the effects of maternal gestation-limited dietary soy on cerebellar postnatal development, motor function, and critical signaling pathways. **Methods:** Pregnant Long Evans rats were fed isocaloric liquid diets containing 0% or 26% caloric ethanol with casein or soy isolate as the protein source. The ethanol and soy feedings were discontinued upon delivery. The offspring were subjected to rotarod motor function tests, and on postnatal day 35, they were sacrificed to harvest cerebella for histological and molecular studies. **Results:** Despite the postnatal cessation of alcohol exposure, chronic gestational exposure reduced brain weight, caused cerebellar hypoplasia, and impaired motor performance. Gestational dietary soy prevented the ethanol-associated reduction in brain weight and largely restored the histological integrity of the cerebellum but failed to normalize motor performance. Ethanol withdrawal abolished the impairments in insulin/IGF signaling that were previously associated with ongoing ethanol exposures, but ethanol’s inhibitory effects on Notch and Wnt signaling persisted. Soy significantly increased cerebellar expression of the insulin and IGF-1 receptors and abrogated several ethanol-associated impairments in Notch and Wnt signaling. **Conclusions:** Although gestation-restricted dietary soy has significant positive effects on neurodevelopment, optimum prevention of FASD’s long-term effects will likely require dietary soy intervention during the critical periods of postnatal development, even after alcohol exposures have ceased.

## 1. Introduction

Fetal Alcohol Spectrum Disorder (FASD) is caused by prenatal alcohol exposure (PAE) via maternal consumption during pregnancy [1,2]. FASD is a clinical diagnosis for which there are no specific biomarkers. A clinical suspicion of FASD is raised based on maternal history and detection of characteristic craniofacial dysmorphic features [3,4,5]. The most significant reasons for recognizing FASD include anticipation, monitoring, and support of neurodevelopmental abnormalities causing cognitive–motor impairments ranging from attention deficit hyperactivity disorders to intellectual disability, the severity of which is proportional to the amount of alcohol consumed by the mother during pregnancy [4,5]. Besides FASD, PAE can cause less severe alcohol-related birth defects and neurodevelopmental disorders with behavioral impairments that can go undetected and, therefore, untreated.

Alcohol exerts neurotoxic effects on many regions and multiple cell types in the immature brain, impairing growth, neuronal survival, plasticity, neuronal migration, and white matter development [6,7]. The cerebellum is particularly vulnerable to the adverse effects of prenatal alcohol exposure. Alcohol’s growth inhibitory effects cause cerebellar hypoplasia [6,8,9] with attendant impairments in motor function [10]. Within the cortex, PAE leads to the loss of Purkinje cells [11,12], which have major roles in coordinated movement, cognition and emotion, loss, hypoplasia, and impaired migration of granule cells [13,14], which receive excitatory input and transmit signals to Purkinje cell dendrites, and deficits in myelination [15]. Cerebellar development including neuronal proliferation, migration, survival, plasticity, and white matter development are largely regulated by insulin/IGF signaling [16,17]. Inhibition of those signaling networks produces morphologic and functional deficits akin to PAE effects [18].

In previous studies, we showed that in experimental animal models, chronic prenatal alcohol exposures leading to FASD were mediated by impairments in placentation and due to the inhibition of signaling through insulin and insulin-like growth factor (IGF) pathways [19]. Consequences include reduced expression and function of aspartyl-asparaginyl-β-hydroxylase (ASPH), which has critical roles in regulating functions such as cell motility and adhesion [20] that are needed for neurodevelopment and plasticity. ASPH’s expression and function are significantly compromised in both the placenta [21] and brain [14] following heavy alcohol consumption in pregnancy. Mechanistically, ASPH activates Notch by catalytic hydroxylation of Asp and Asn residues in EGF-like domains [22,23]. Activated Notch upregulates the transcription of hairy and enhancer of split-1 (HES-1) and related molecules [22,23] needed to regulate diverse developmental functions such as growth and maturation [24]. In addition, the Wnt pathway has demonstrated roles in neurodevelopment [25] and cross-talks with insulin/IGF [26,27] and Notch pathways [28,29]. The inhibitory effects of ethanol on insulin/IGF, Notch, and Wnt pathway signaling in the placenta and brain have been well documented [21,23].

Public health preventive measures are needed to more effectively consider the big picture related to the maternal, placental, and fetal effects of prenatal alcohol exposures and their long-term consequences in the offspring [30]. Ideally, approaches should take advantage of knowledge gained from basic and translational research. Given the importance of insulin/IGF, Notch, and Wnt networks during development and the considerable array of functional deficits that result from impairments in signal transduction and gene expression, efforts should be made to fortify the integrity of these pathways in the context of prenatal and perinatal alcohol exposures. Basic and translational research led to the concept that insulin sensitizers that also provide antioxidant and anti-inflammatory support could address problems stemming from prenatal alcohol exposures, including FASD. To this end, we evaluated the potential therapeutic effects of small molecule peroxisome proliferator-activated receptor (PPAR) agonists, dietary soy, and bioactive soy constituents. Previous studies showed that insulin sensitizer interventions could prevent or reduce the adverse CNS effects of insulin resistance caused by chronic alcohol feeding, high-fat diet-induced obesity, or nitrosamine administration [31,32,33]. However, since the application of PPAR agonist therapy to pregnant women is limited due to unpredictable harm across the maternal–placental–fetal axis, we turned our attention to the potential beneficial effects of natural insulin-sensitizing substances such as soy or its bioactive isoflavones, daidzein, and genistein [34]. Correspondingly, in recent studies, we demonstrated that chronic feeding with dietary soy minimized or prevented the adverse effects of prenatal alcohol exposures on placentation and fetal development [19], and that later adolescent exposures also positively impacted cognitive–behavioral function [35]. However, our follow-up question was whether dietary soy administered only in pregnancy would suffice for preventing FASD-related neurodevelopmental pathologies that emerged postnatally. The working hypothesis was that following prenatal alcohol exposure, dietary soy’s rescue or preventive benefits on cerebellar neurodevelopment and maturation would be suboptimum or incomplete if not provided throughout the critical period. The cerebellum was selected for study because it is a major target of alcohol-related neurotoxicity, and it develops from the early prenatal period (from embryonic day 8.5) to the first three weeks of postnatal life in rodents [36]. The present study extends our previous work by generating gestational FASD models and examining the effects of discontinuing chronic alcohol exposures and dietary soy feeding at birth. The experimental paradigm provided an opportunity to assess the potential need for continuous dietary soy support across the full span of neurodevelopment and maturation following prenatal alcohol exposure. Postnatal brain development, neurobehavioral function, and insulin/IGF, Notch, and Wnt pathway signaling molecule expression were evaluated to characterize potential mediators of sustained recovery or persistence of FASD features vis à vis early postnatal ethanol and dietary soy withdrawal in the offspring. It is noteworthy that the study design was focused on the offspring and not maternal effects which were evaluated in earlier studies [19,37].

## 2. Materials and Methods

### 2.1. Study Design Overview

This is a preclinical study in which an established prenatal alcohol exposure (PAE) model was used to better characterize the potential benefits of dietary soy for preventing neurodevelopmental abnormalities associated with FASD. The research focused on the cerebellum because of its high vulnerability to the adverse effects of PAE and attendant contributions to motor and cognitive dysfunction. Previous studies showed that feeding pregnant dams with soy protein isolate instead of casein prevented many of the cranial-facial and neurodevelopmental abnormalities in the term fetuses [19]. However, we did not determine if subsequent brain development would be normalized in the absence of continued exposure to dietary soy. This question is particularly relevant to the cerebellum, which continues to grow and mature during the postnatal period. To address this point, the study was designed using a four-way model in which pregnant dams were fed control or ethanol-containing liquid diets with either casein (standard) or soy isolate (experimental) provided as the sole source of protein. The offspring rats were assessed for cerebellar motor function and image analysis for changes in neuronal populations, and cerebellar tissue was analyzed using molecular and biochemical approaches to evaluate the integrity of signaling pathways known to be important for neurodevelopment and impaired by chronic alcohol exposure.

### 2.2. Materials

Qiazol EZ1 RNA kit, QuantiTect SYBR Green polymerase chain reaction (PCR) master mix, and the BIO Robot Z1 were purchased from Qiagen Sciences, Inc. (Valencia, CA, USA). The Akt Pathway Total and Phospho Multiplex panels were purchased from Invitrogen (Carlsbad, CA, USA) (Supplementary Table S1). The AMV 1st Strand cDNA Synthesis Kit and the reagents and probes for qPCR were purchased from Roche Applied Science (Indianapolis, IN, USA) (Supplementary Table S2). The bicinchoninic acid (BCA) kit to measure protein concentration was purchased from Pierce Chemical Co (Rockford, IL, USA). Histochoice fixative was purchased from Amresco, Inc. (Solon, OH, USA). Fine chemicals were purchased from CalBiochem (Carlsbad, CA, USA) or Sigma-Aldrich (St. Louis, MO, USA).

### 2.3. Experimental Model

The experimental model was generated as previously described [19]. In brief, adult Long Evans female and male rats (purchased from Charles River Laboratories; Willmington, MA, USA) were mated in our facilities by placing two males with one female in a cage and performing daily morning vaginal swabs to detect evidence of the first mating. Gestation day 0 (GD0) was designated as the morning in which sperm was detected in metestrus stage vaginal smears [38], after which the males were removed from the cages. The males and females were mated only once. The pregnant dams were fed with isocaloric liquid diets (BioServ, Frenchtown, NJ, USA) that contained 0% (control) or 26% pharmaceutical grade ethanol (caloric content) with either casein (standard, control) or soy isolate as the protein source (see Supplementary Table S1 for the dietary nutritional compositions). The soy protein isolate was not alcohol-stripped and therefore likely contained 0.6 mg to 1.64 mg isoflavone/g protein [39,40]. The liquid diets were initiated on GD 6 to minimize the adverse effects of alcohol exposure on implantation. The resulting four experimental groups were designated as (1) CC for control-casein; (2) CS, control-soy; (3) EC for ethanol-casein; and (4) ES for ethanol-soy, including 4 pregnant dams per group.

The liquid diets were continued until postnatal day 0 (P0; delivery), after which all diets were switched to chow (see Supplementary Table S1A,B for nutritional composition). Each dam was housed in a separate cage with its litter. All pups were ear-tagged to track growth, behavioral performance, and studies performed on their harvested cerebella. The pups were weighed at birth and then weekly. The weaned offspring were housed in pairs by sex (not litter of origin), and they were maintained on chow ad libitum with free access to water. The animal facility was pathogen-free and equipped with an automated 12 h light/dark cycle (lights on at 7:00 a.m. and lights off at 7:00 p.m.). Enrichment toys were not used due to potential differential effects on neurobehavioral performance in alcohol exposure models. Rotarod testing was performed on P16. On P35 (5 Weeks), the rats were sacrificed by isoflurane inhalation followed by cutting the diaphragm to create a pneumothorax. After harvesting the brains, the cerebella were hemisected for immersion fixed in 10% neutral buffered formalin and processed by paraffin embedding or snap freezing on dry ice and storage at −80 °C for molecular and biochemical studies. Formalin-fixed paraffin-embedded histological sections (5 µm thick) were stained with Luxol fast blue/hematoxylin and eosin (LHE) to reveal neuronal and glial cytomorphology and white matter fibers. The study was conducted according to the guidelines of the Declaration of Helsinki and approved by the Institutional Animal Care and Use Committee (IACUC) at Rhode Island Hospital and Lifespan (Committee #503823 approved 28 September 2023), and the protocol adheres to the National Institutes of Health (NIH) Guide for the Care and Use of Laboratory Animals.

### 2.4. Rotarod Testing

Rotarod testing was used to assess cerebellar motor function. On P16, rats were administered ten incremental speed rotarod trials from 1.5 to 6.0 rpm using a Rotamex-5 (Columbus Instruments, Columbus, OH, USA), allowing 10 min of rest between trials. All rats were tested in each of the ten trials. The latencies to fall were automatically recorded with photocells placed over the rod. The maximum duration of work was capped at 45 s, after which the trials were stopped to prevent exercise fatigue. Therefore, the scores ranged from 1 to 45 s. Results from Trials 1–3 (1.5 to 2.5 rpm)—low speed, 4–7 (3.0 to 5.0 rpm)—medium speed, and 8–10 (6.0 rpm)—high speed were culled and analyzed with the Mann–Whitney test.

### 2.5. Stereology

Unbiased stereology was performed with Stereologer software (Stereology Resources, Inc., Chester, MD, USA, available on https://srcbiosciences.com/stereologer-software accessed on 24 February 2025) to quantify the effects of ethanol and dietary soy on cerebellar pathology. The measurements were focused on determining the number of granule and Purkinje cells per volume of cortical gray matter. The Volume probe was selected to measure gray matter, and the two Disector probes assessed granule and Purkinje cell abundance. Volumes were measured at 4× using the area point-count method with a distance between x’s at 5% of the screen area. Both dissectors were measured at 40×. ThePurkinje cells were counted using a frame size of 40% of the screen height, whereas the granule cells were counted using a frame size that was 0.5% of the screen height. The distance between dissectors was entered as 35 µm whereas the dissector height and guard height were 7 µm and 2 µm, respectively.

### 2.6. Sample Processing for Molecular and Biochemical Studies

For molecular studies of immunoreactivity and mRNA, samples from all litters and both sexes were included in the analyses. To minimize litter effects, a technician with no knowledge of the neurobehavioral performance or other features of the offspring selected two samples from each of the four CC, EC, CS, and ES groups to be included in each of the four major assays (Akt pathway total and phospho-ELISAs, Notch PCR, Wnt PCR) [41,42]. A total of 8 samples were analyzed per treatment/diet group per assay. Results were analyzed using mixed models ANOVA and post hoc Tukey multiple comparisons tests. The sample sizes were based on power analysis calculations using preliminary and prior data. The study design included both sexes but lacked sufficient power to analyze sex as a biological variable. For the immunoassays, hemi-cerebella tissue samples (100 mg) were homogenized in 5 volumes of Weak Lysis Buffer (150 mM NaCl, 50 mM Tris-Base pH 7.5, 0.1% Triton X-100, 5 mM EDTA pH 8.0, 10 mM EDTA, 50 mM NaF) that contained a protease inhibitor cocktail and phosphatase inhibitors as described above. Supernatant fractions obtained after centrifuging the samples at 14,000× *g* for 15 min at 4 °C were used in the immunoassays.

### 2.7. Multiplex Enzyme-Linked Immunosorbent Assay (ELISA)

Magnetic multiplex bead-based assay kits from Invitrogen (Carlsbad, CA USA) measured total AKT (Catalog #LHO0002M) and phospho-AKT (Catalog #LHO0001M) insulin/IGF/IRS-Akt pathway molecules following the manufacturer’s protocol (Supplementary Table S1). Samples containing 100 μg protein/sample were incubated with the beads. The captured antigens were detected with biotinylated secondary antibodies and phycoerythrin-conjugated Streptavidin. Immunoreactivity was measured in a MAGPIX with xPONENT Version 4.3 software. Standard curves generated with known concentration ranges of each analyte were used to calculate the levels of immunoreactivity in the samples. Negative control studies included reactions devoid of brain tissue homogenates. The results are expressed as arbitrary fluorescence light units (FLUs) calculated from standard curves.

### 2.8. Duplex Quantitative Reverse Transcriptase Polymerase Chain Reaction (qRT-PCR) Analysis

Quantitative reverse transcriptase polymerase chain reaction (qRT-PCR) analysis was used to measure Notch pathway mRNA transcripts, including aspartyl-asparaginyl-β-hydroxylase (ASPH), Notch 1, Jagged1, hairy/enhancer of split-1 (HES1), hypoxia-inducible factor-1 (HIF-1α), factor inducing hypoxia (FIH), and selected Wnt canonical and non-canonical pathway mRNA transcripts, including Wnt5a, Wnt5b, Frizzled 4 (Fzd4), Fzd6, E1a Binding Protein 300 (EP300), Dixdc1, and Axin2 RNA. RNA extracted from fresh frozen tissue using the RNeasy Mini Kit (Qiagen, Valencia, CA, USA) was reverse-transcribed with random oligonucleotide primers and the AMV 1st Strand cDNA Synthesis Kit. Gene expression was measured using TaqMan hydrolysis probe-based duplex qRT-PCR assays in which β-actin served as a reference gene [22]. Reactions (20 μL) contained Taqman Gene Expression Master Mix, 200nM of gene-specific and β-actin primers, 100 nM of β-actin (Y555-labeled), and gene of interest (FAM-labeled) probes (Supplementary Table S2). The ProbeFinder software 2.0 (Roche, Indianapolis, IN, USA) determined gene-specific primer sequences and matched probes (Supplementary Table S3). PCR amplifications were initiated by a 10 min, 95 °C denaturation step and followed by 45 2-step cycles of denaturation (15 s at 95 °C) and annealing/extension (1 min at 60 °C). The PCR amplifications were performed in a LightCycler 480 PCR machine (Roche, Indianapolis, IN, USA). Fluorescence signals corresponding to the genes of interest were acquired in the FAM channel (Em: 483–533 nm), and the β-actin signal was acquired in the Y555/HEX channel (Em: 523–568 nm). Results were analyzed using LightCycler software 4.0.

### 2.9. Statistical Analysis

Neurobehavioral testing, image analysis, and molecular data acquisition were performed under code. Statistical analysis was performed, and graphs were generated using the GraphPad Prism 10.2 software (GraphPad Software, Inc., San Diego, CA, USA) which determined the data to be normally distributed. Inter-group comparisons were made using mixed model analysis of variance (ANOVA) with the post hoc Tukey multiple comparisons test of significance. Statistical significance was set at *p* ≤ 0.05. Statistical trends defined as 0.05 < *p* < 0.10 were also noted. Initial studies demonstrated a lack of sex-specific effects on the molecular/biochemical assay results. - Moreover, since the study was not sufficiently powered to investigate sex as a biological variable, the data from both sexes were combined for inter-group statistical comparisons.

## 3. Results

### 3.1. Growth and Brain Weight

**Figure 1:**
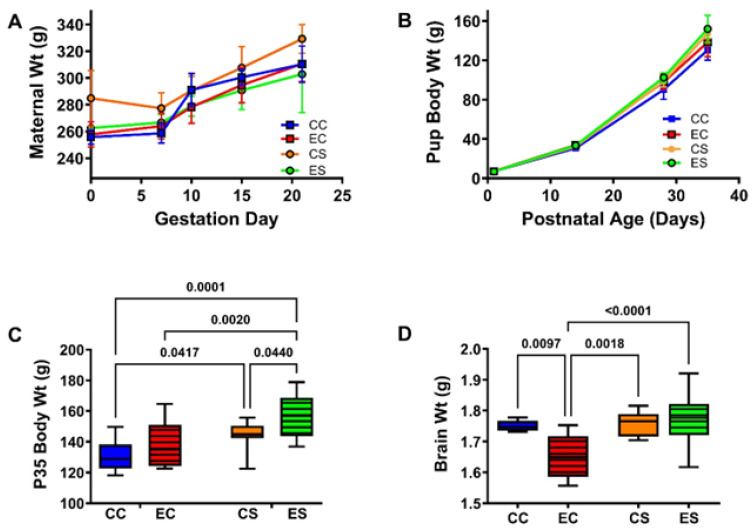
Maternal and pup body weight gains and final brain weight in the offspring. (**A**) Weight gain (mean ± S.D.) trajectories in the pregnant dams. Post hoc test of the two-way ANOVA demonstrated no significant intergroup differences in maternal body weights. (**B**) Pups were weighed at birth and weekly. There were no significant intergroup differences in the mean pup birth weights by two-way ANOVA (Table 1 and Supplementary Table S4). Experimental endpoint mean (**C**) body and (**D**) brain weights are represented with box plots such that the dense horizontal bars represent the median, the upper and lower boundaries of the boxes reflect 95% confidence limits, and the stems correspond to range. The data were analyzed by two-way ANOVA. The significant post hoc test results are shown in the panels. See Supplementary Table S4 for detailed comparisons of dams’ G0 and G21 body weights and the offsprings P0 and P35 body weights and P35 brain weights. Abbreviations: CC = control-casein; EC = ethanol-casein; CS = control-soy; ES = ethanol-soy.

The pregnant dams in all groups progressively gained weight (Figure 1A). Although the CS dams tended to weigh more than rats in the other three groups, no significant inter-group differences were detected with respect to the mean body weights measured at GD0 or on the day of delivery (GD21/P0; Table 1 and Supplementary Table S4). The total number of pups generated in each group varied between 25 and 41. Chi-square analysis demonstrated no significant differences with respect to the proportions of males and females in each group (χ^2^ = 2.31, N.S.) (Supplementary Table S4). There were no significant inter-group differences in mean birth weight (Table 1 and Supplementary Table S4), and all pup groups continuously gained weight over the time course of the study (Figure 1B). In contrast, significant intergroup differences in mean body weight measured at P35, the experimental endpoint, were detected by two-way ANOVA (Table 1 and Supplementary Table S4). The Tukey post hoc multiple comparisons test demonstrated significantly higher mean body weights in the dietary soy-fed relative to casein-fed groups, with the largest effect in the ES group (Figure 1C and Supplementary Table S4). The terminal (P35) mean brain weights also significantly varied among the groups (Supplementary Table S4 and Table 1) due to a significant reduction in EC relative to the other three groups (Figure 1D and Supplementary Table S4). Of note is that the mean brain weight in the ES rats was normalized and did not differ significantly from the CC or CS (Supplementary Table S4).

### 3.2. Neurobehavioral Testing

**Figure 2:**
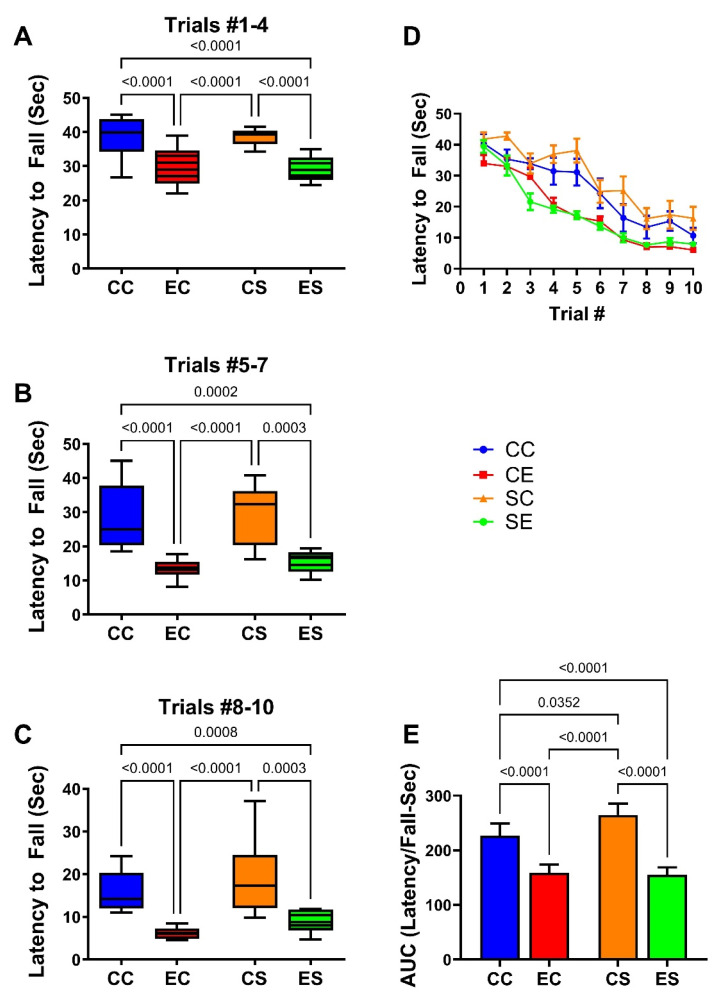
Rotarod performance. Rotarod performance was measured over 10 consecutive trials of increasing rod rotation speed. The latencies to fall off the rod (seconds) were optically recorded. Results were culled into three groups: (**A**) trials 1–4 represent low rotation speeds (1.5–3.0 rpm); (**B**) trials 5–7 correspond to moderate speed (3.5 and 5.0 rpm); (**C**) trials 8–10 represent the highest speeds (6.0–8.0 rpm). (**D**) Declining performance (latency to fall) (mean ± S.D.). (**E**) Mean ± S.D. of the area-under-curve calculations of results used to generate graphs in (**D**). Results were analyzed by two-way ANOVA. Significant inter-group differences detected with post hoc Tukey multiple comparisons tests are depicted in the panels.

The rotarod test was used to assess cerebellar function. Performance was assessed with progressively increasing rod rotation speed. The maximum latency was capped at 45 s to prevent fatigue. The results were analyzed by culling the latency to fall responses over Trials 1–4 (low speed; Figure 1A), 5–7 (moderate speed; Figure 1B), and 8–10 (high speed; Figure 2C), plotting the mean group performance for each trial (Figure 2D) and calculating the area-under-curve (AUC) for overall performance in Trials 1–10 (Figure 2E). Summary results reflecting the mean latency to fall (in seconds) revealed similar performance in the CC and CS groups with reductions in the mean latencies from low- to moderate- to high-speed rod rotations (Figure 2A–C). The EC and ES groups exhibited similarly reduced mean latencies to fall in each of the clustered trials with significant differences from both CC and CS control groups. In essence, EC and ES had similarly impaired performance relative to CC and CS groups in the low-speed (Figure 2A), moderate-speed (Figure 2B), and high-speed (Figure 2C) trials. The linear graphs reflecting performance for each trial demonstrated similarly consistent reductions in mean latency to fall in the EC and ES groups over the entire study (Figure 2D). Correspondingly, AUC analysis of those curves revealed similar degrees of impairment in the EC and ES groups (Figure 2E).

### 3.3. Cerebellar Pathology

**Figure 3:**
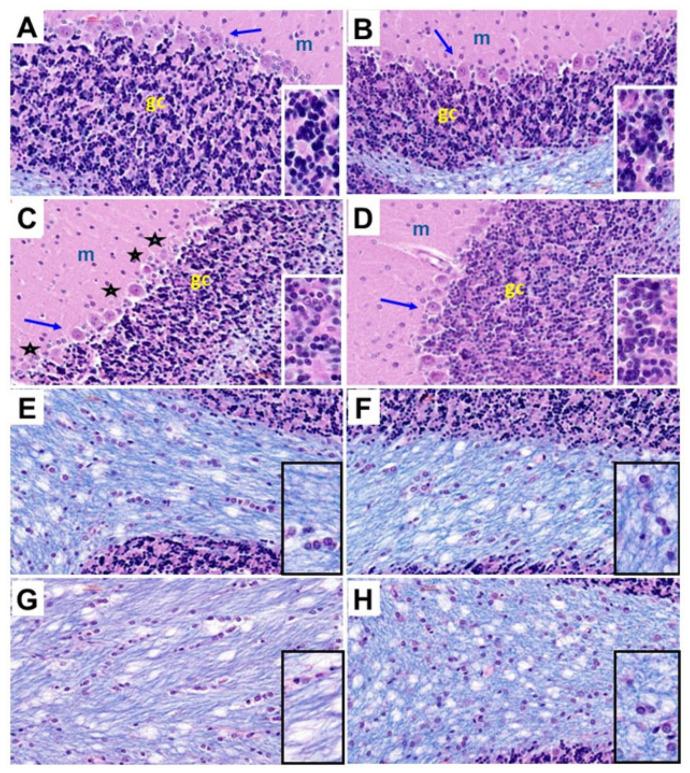
Ethanol and dietary soy effects on cerebellar histology. Histological sections of (**A**–**D**) cerebellar cortex and (**E**–**H**) subcortical white matter from (**A**,**E**) CC, (**B**,**F**), CS, (**C**,**G**) EC, and (**D**,**H**) ES rats harvested on P30. The paraffin-embedded histological sections were stained with Luxol fast blue and hematoxylin and eosin. Three cortical layers characteristic of cerebellum, including molecular (m), Purkinje (arrows), and granule cell (gc) layers are shown with higher magnification images of the gc layers shown in the insets. EC cortical structure depicts variability and loss of Purkinje cells (asterisks) and reduced density of granule cells (dark blue-black nuclei in the insets). ES cortical structure is similar to CC and CS. (**E**–**H**) White matter myelinated fibers stain blue with Luxol fast blue. Small, scattered nuclei mainly represent glial cells. Edges of cortical granule cells are shown in (**E**,**F**,**H**). The density of myelin staining and distribution of glial cells were similar in CC, CS, and ES samples. (**G**) EC cerebellar white matter exhibits pallor (less intense blue staining and larger vacuoles (clear areas). (**H**) Glial cell density may be increased in ES white matter. All images were photographed at 400×. Insets correspond to approximately 900× magnification.

Histological sections of the cerebellar cortex and underlying white matter stained with hematoxylin and eosin (H&E) and Luxol fast blue (LFB) compared the effects of chronic gestational ethanol exposure and maternal dietary soy on cerebellar cortical architecture and cellularity (H&E) (Figure 3A–D) and myelin integrity (LFB) (Figure 3E–G). Control cerebella from the CC (Figure 3A) and CS (Figure 3B) groups had abundant populations of granule cells and Purkinje cells. Granule cells were tightly packed, and Purkinje cells uniformly lined the zone between molecular and granule cell layers. Chronic ethanol exposure in the EC dams reduced the density of granule cells and resulted in neuronal loss and atrophy in the Purkinje cell layer (Figure 3C). The inclusion of soy in the diets somewhat increased the density of granule cells and rendered the Purkinje cell layer more uniform (Figure 3D). White matter myelin stained with LFB exhibited a blue tint whereas the glial and endothelial cells were stained with H&E. CC (Figure 3E) and CS (Figure 3F) white matter exhibited similar intensities and uniformities of LFB staining and densities of glial cell elements. In contrast, chronic ethanol exposure reduced the intensity of white matter LFB staining and enlarged the spacing (cleared areas separating myelin fibrils), i.e., vacuolation (Figure 3G). Dietary soy was associated with increased intensity of white matter LFB staining relative to EC but also increased the apparent density of glial cells relative to CC and CS (Figure 3).

### 3.4. Cerebellar Image Analysis

**Figure 4:**
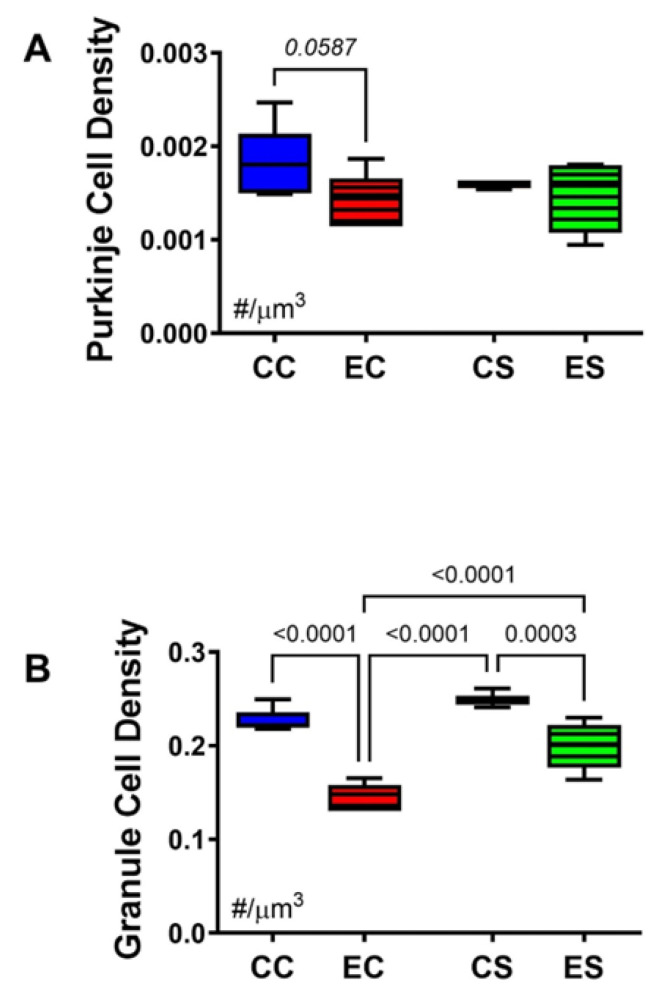
Cerebellar morphometrics. Histological sections of cerebellar cortex were subjected to image analysis to evaluate the effects of chronic gestational alcohol exposure and dietary soy on Purkinje and granule cell densities. Numerical densities of (**A**) Purkinje and (**B**) granule cells were quantified per cubic micron of cortical gray matter present on the slide with the aid of an Olympus BX60 light microscope (Olympus America Inc., Center Valley, PA, USA) with an attached MS-2000 XYZ Inverted Stage (Applied Scientific Instrumentation, Eugene, OR, USA) and Stereologer software (Stereology Resource Center, Inc., Chester, MD, USA). Unbiased counting frames were applied under software control. Cells were counted at high magnification (40×) using optical dissector method and normalized to the volume which was based on Cavalieri point grid method. The results were analyzed by two-way ANOVA. Significant (*p* ≤ 0.05) and statistical trendwise (0.05 < *p* < 0.10; italics) differences detected with post hoc Tukey multiple comparisons tests are displayed within the panels.

**Figure 5:**
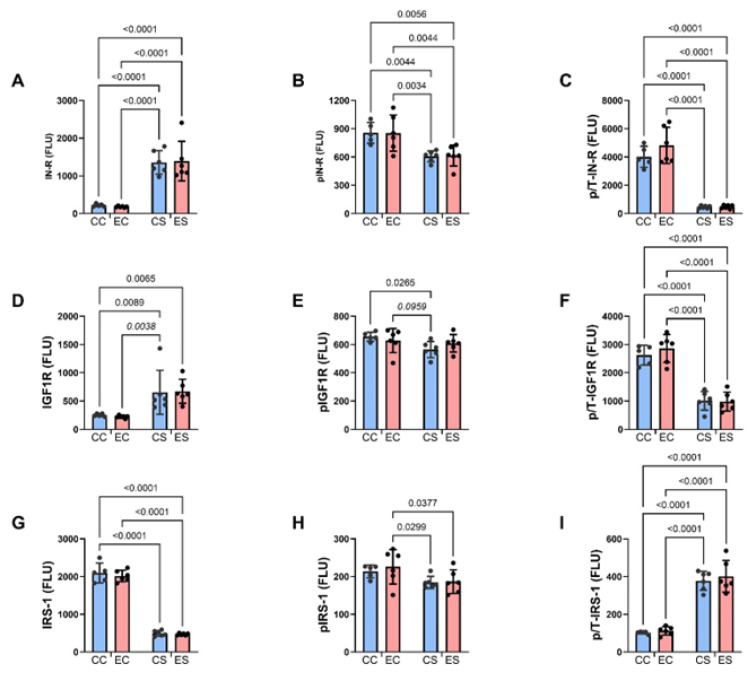
Upstream insulin and insulin-like growth factor signaling molecules. Cerebellar homogenates were used to measure (**A**) insulin receptor (IN-R), (**D**) IGF-1R, (**G**) IRS-1, (**B**) pYpY1162/1163- IN-R, (**E**) pYpY1135/1136-IGF-1R, and (**H**) pS312-IRS-1 with the Total and Phospho Akt 7-Plex Panels. Relative levels of phosphorylation are represented by the calculated ratios of (**C**) pYpY1162/1163-/IN-R, (**F**) pYpY1135/1136-/IGF-1R, and (**I**) pS312/IRS-1. N = 8 in each of the CC, EC, CS, and ES groups. See summary results in Table 3. Significant (*p* ≤ 0.05) and statistical trendwise (0.05 < *p* < 0.10; italics) differences detected with post hoc tests are displayed within the panels.

Stereological analysis was used to calculate the densities of Purkinje (Figure 4A) and granule (Figure 4B) cells in the cerebellar cortex. One-way ANOVA detected a statistical trendwise effect for intergroup differences in Purkinje cell densities (F = 3.327; *p* = 0.0587) and statistically significant differences for granule cell densities (F = 45.64; *p* < 0.0001). Post hoc tests revealed a trend reduction in Purkinje cell density in EC relative to CC cerebella (Figure 4A) but otherwise similar mean densities of Purkinje cells in the cerebellar cortex of the CC, CS, and ES groups. In contrast, the density of granule cells was significantly reduced in both EC and ES relative to both control groups, i.e., CC and CS (Figure 5B). However, the mean density of cortical granule cells was significantly higher in the ES compared with EC samples. In essence, chronic gestational exposure to ethanol reduced the densities of Purkinje and granule cells in the cerebellar cortex, but dietary supplementation with soy largely prevented the cell loss or enabled its recovery relative to EC.

### 3.5. Multiplex Immunoassays of Insulin Receptor/IGF-1 Receptor/IRS-1 Pathway

Previous studies showed that chronic ethanol exposures lead to impairments of insulin and IGF-1 signaling through IRS and Akt pathways and correlated those effects with alcohol-related brain pathology and neurobehavioral dysfunctions [43]. To examine the extent to which dietary soy’s therapeutic or rescue effects in the FASD model were mediated by restoration of these signaling networks, the cerebellar tissue samples were analyzed with multiplex total and phosphoprotein ELISAs. In addition, the calculated relative levels of protein phosphorylation were assessed. Two-way ANOVA detected significant dietary protein source effects on the expression of the insulin receptor, IGF-1 receptor, IRS-1, GSK-3β, pY-Insulin receptor, pY-IGF-1 receptor, pS-IRS-1, pS-Akt, and the relative phosphorylation levels (p/T) of the insulin and IGF-1 receptors, IRS-1 and Akt, but not Akt, pS-GSK-3β, or pS/T-GSK-3β (Table 2). No significant independent effects of ethanol or diet × ethanol interactions were observed.

**Table 2:** Insulin/IGF-1, IRS1-Akt pathway (ELISA)—ANOVA tests.

Molecule	Diet F-Ratio	*p*-Value	Ethanol F-Ratio	*p*-Value	Interaction F-Ratio	*p*-Value
**Insulin R**	**79.95**	**<0.0001**	0.0002	0.989	0.078	0.784
**IGF-1R**	**20.10**	**0.0003**	0.003	0.954	0.0689	0.796
**IRS-1**	**642.9**	**<0.0001**	0.751	0.397	0.193	0.665
**Akt**	0.538	0.472	0.188	0.670	2.535	0.128
**GSK-3** **β**	**18.61**	**0.0004**	0.716	0.408	1.65	0.214
**pY-Insulin R**	**20.86**	**0.0002**	0.002	0.961	0.011	0.919
**pY-IGF-1R**	**4.417**	**0.049**	0.0998	0.756	1.902	0.184
**pS-IRS-1**	**7.140**	**0.015**	0.322	0.577	0.169	0.686
**pS-Akt**	**10.97**	**0.0037**	0.119	0.742	0.947	0.343
**pS-GSK-3** **β**	1.574	0.225	0.672	0.423	0.565	0.462
**p/T-Insulin R**	**160.5**	**<0.0001**	1.731	0.204	1.659	0.213
**p/T-IGF-1 R**	**118.1**	**<0.0001**	0.395	0.537	0.674	0.422.
**p/T-IRS-1**	**170.3**	**<0.0001**	0.584	0.454	0.096	0.761
**p/T-Akt**	**13.75**	**0.0015**	0.031	0.862	0.068	0.798
**p/T-GSK-3** **β**	0.363	0.554	0.455	0.515	0.922	0.349

**Figure 6:**
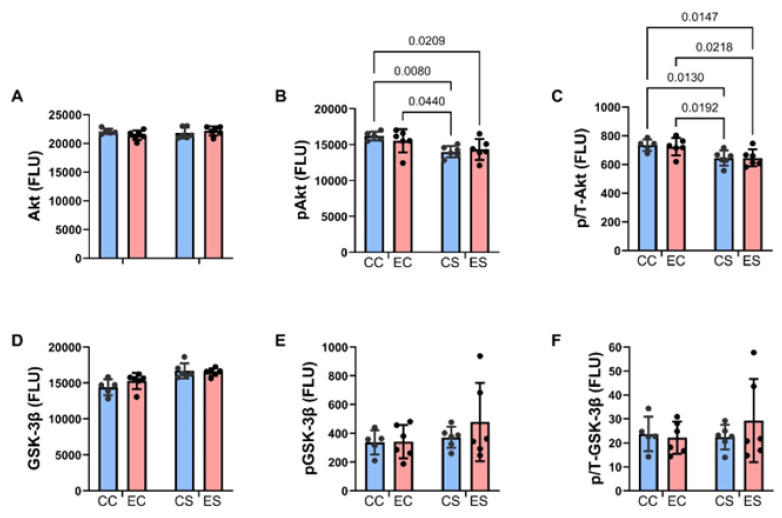
Effects of chronic gestational exposures to ethanol and dietary soy on downstream insulin and IGF pathway signaling. Cerebellar homogenates were used to measure (**A**) Akt, (**D**) GSK-3β, (**B**) pS473-Akt, and (**E**) pS9-GSK-3β, with the Total and Phospho Akt 7-Plex Panels. Relative degrees of phosphorylation are shown by the calculated ratios of (**C**) pS473-/Akt and (**F**) pS9-/GSK-3β. N = 8 in each of the CC, EC, CS, and ES groups. See summary results in Table 3. Significant (*p* ≤ 0.05) inter-group differences detected with post hoc tests are displayed within the panels.

Immunoreactivity was measured with an Akt pathway magnetic bead-based panel. The table lists the two-way ANOVA test results (F-ratios and *p*-values). For all tests: diet factor (soy or casein as the protein source), ethanol factor, and diet × ethanol interaction DFn, DFd (1, 28). Bold font highlights significant results (*p* ≤ 0.05). N = 8 rats/group. See Figure 5 and Figure 6 for corresponding graphs and post hoc test results. Abbreviations: R = receptor; IGF = insulin-like growth factor; IRS = insulin receptor substrate; GSK-3β = glycogen synthase kinase-3β; pY = tyrosine phosphorylated; pS = serine phosphorylated.

**Table 3:** ASPH–Notch network (mRNA)-ANOVA tests.

Molecule	Diet F-Ratio	*p*-Value	Ethanol F-Ratio	*p*-Value	Interaction F-Ratio	*p*-Value
**ASPH**	0.792	0.384	2.199	0.161	0.959	0.339
**NOTCH 1**	2.044	0.168	0.041	0.842	0.000	0.980
**JAGGED 1**	**5.931**	**0.024**	1.428	0.246	**5.218**	**0.033**
**HES1**	1.100	0.307	1.251	0.277	*3.603*	*0.072*
**HIF-1α**	2.611	0.122	**4.660**	**0.043**	**6.404**	**0.0199**
**FIH**	**4.374**	**0.049**	0.249	0.623	**9.646**	**0.0056**

**Figure 7:**
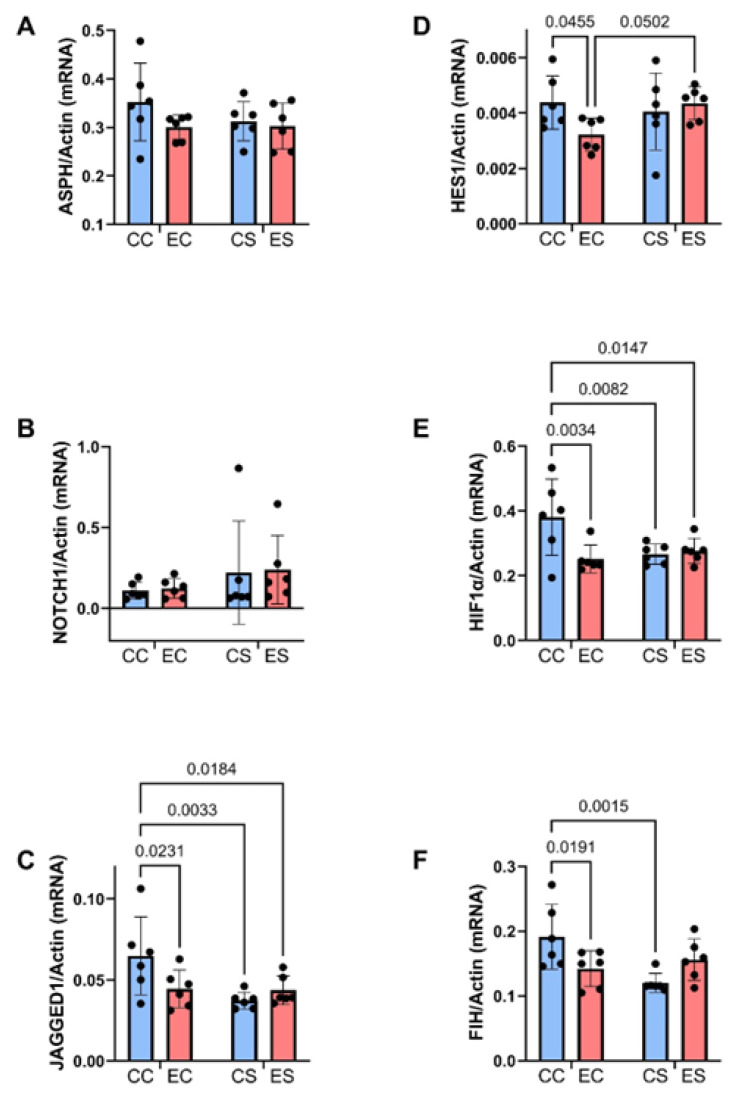
Ethanol and dietary soy effects on Notch signaling networks. Gene expression was measured by duplex qRT-PCR analysis with a probe-hydrolysis detection system. The b-actin housekeeping gene was simultaneously amplified and detected in the same well as the gene of interest (see Methods). Graphs depict relative mRNA abundance (mean ± S.D.) of (**A**) ASPH, (**B**) Notch1, (**C**) Jagged1, (**D**) HES1, (**E**) HIF-1α, and (**F**) FIH. Two-way ANOVA made inter-group comparisons (see Table 4) with post hoc Tukey tests. Significant (*p* ≤ 0.05) inter-group differences detected with post hoc tests are displayed within the panels.

The mRNA levels were measured with a duplex PCR (Taqman-based) assay with results normalized to actin measured in the same samples. The table lists the two-way ANOVA test results (F-ratios and *p*-values). For all tests: diet factor, ethanol factor, and diet × ethanol interaction DFn, DFd (1, 28). Bold font highlights significant results (*p* ≤ 0.05). Statistical trendwise differences (0.05 < *p* < 0.10) are italicized. N = 8 rats/group. See Figure 7 for corresponding graphs and post hoc test results. Abbreviations: ASPH = aspartyl-asparaginyl-β-hydroxylase; HES1 = hairy and enhancer of split-1; HIF-1α = hypoxia inducible factor 1α; FIH = factor inhibiting hypoxia-inducible factor 1.

Bar graphs display the effects of ethanol and dietary soy on insulin/IGF-1/IRS-1-Akt pathway proteins and phosphoproteins (Figure 5 and Figure 6). Regarding the upstream components of the pathway, the sources of significant variance detected by two-way ANOVA tests were due to similarly elevated levels of insulin receptors (Figure 5A), IGF-1 receptors (Figure 5D), and p/T IRS-1 (Figure 5I), and reduced expression of pY-Insulin receptors (Figure 5B), p/T Insulin (Figure 5C) and IGF-1 (Figure 5F) receptors, and IRS-1 (Figure 5G) in CS and ES relative to CC and/or EC. In addition, pS-IRS-1, which is inhibitory to IRS-1 signaling [44] was significantly reduced in CS and ES relative to EC (Figure 5H). Regarding downstream signaling through Akt and GSK-3β, pS-Akt (Figure 6B) and p/T-Akt (Figure 6C) were significantly reduced in CS and ES relative to CC and/or CE, indicating soy-medicated reductions in Akt signaling, irrespective of ethanol exposure. In contrast, there were no significant inter-group differences in the levels of total Akt (Figure 6A), total GSK-3β (Figure 6D), pS-GSK-3β (Figure 6E), or p/T-GSK-3β (Figure 6F).

**Table 4:** Wnt pathway (mRNA)-ANOVA tests.

Molecule	Diet F-Ratio	*p*-Value	Ethanol F-Ratio	*p*-Value	Interaction F-Ratio	*p*-Value
**Wnt 5a**	**5.263**	**0.0327**	**7.269**	**0.0139**	**8.424**	**0.0088**
**Wnt 5b**	*4.05*	*0.0571*	2.685	0.1169	**5.063**	**0.0359**
**Fzd 4**	**5.418**	**0.0305**	0.99	0.33	0.006	0.937
**Fzd 6**	**5.702**	**0.0269**	**8.044**	**0.0102**	**7.776**	**0.0113**
**EP300**	0.060	0.808	**5.788**	**0.0259**	0.449	0.510
**Dixdc1**	1.194	0.287	*3.242*	*0.0869*	**11.59**	**0.0028**
**Axin2**	2.563	0.125	**6.065**	**0.023**	**5.024**	**0.0365**

**Figure 8:**
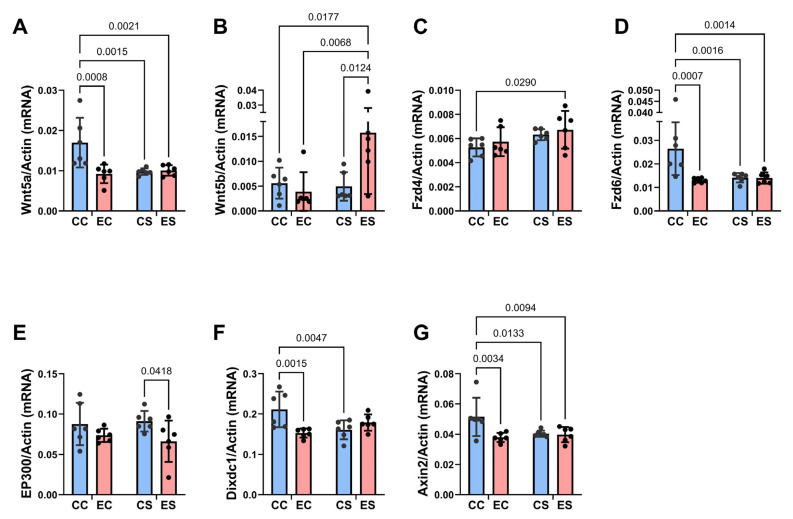
Ethanol and soy effects on Wnt signaling networks. RNA was isolated from fresh frozen cerebellar tissue, and mRNA expression was measured by duplex qRT-PCR analysis with a probe-hydrolysis detection system. β-actin was amplified and detected in the same well as the gene of interest (see Methods). Graphs depict relative mRNA abundance (mean ± S.D.) of (**A**) Wnt5a, (**B**) Wnt5b, (**C**) FZD4, (**D**) FZD6, (**E**) EP300, (**F**) Dixdc1, and (**G**) Axin2. Two-way ANOVA and post hoc Tukey tests were used for inter-group comparisons (see Table 4). Significant (*p* ≤ 0.05) inter-group differences detected with post hoc tests are displayed within the panels.

The mRNA levels were measured with a duplex PCR (TaqMan-based) assay with results normalized to actin measured in the same samples. The table lists the two-way ANOVA test results (F-ratios and *p*-values). For all tests: diet factor, ethanol factor, and diet × ethanol interaction DFn, DFd (1, 28). Bold font highlights significant results (*p* ≤ 0.05). Statistical trendwise differences (0.05 < *p* < 0.10) are italicized. N = 8 rats/group. See Figure 8 for corresponding graphs and post hoc test results. Abbreviations: Fzd = Frizzled; EP300 = E1A binding protein p300; Dixdc1 = Disheveled-Axin domain containing-1.

### 3.6. Notch Pathway mRNA Studies

Previous studies linked the adverse effects of prenatal alcohol exposure on cerebellar development to impairments in signaling through the Notch pathway, including expression of aspartyl-asparaginyl-β-hydroxylase (ASPH) [35,45]. Using duplex RT-PCR assays with actin as the loading control, the relative levels of ASPH, Notch1, Jagged1, HES-1, HIF-1α, and FIH were measured in cerebellar tissue. Two-way ANOVA tests detected significant dietary protein effects on Jagged1 and FIH expression, ethanol effects on HIF-1α expression, and dietary protein × ethanol interactive effects on Jagged 1, HIF-1α, and FIH, and a statistical trend interactive effect of dietary protein × ethanol on HIF-1α (Table 3). The post hoc tests demonstrated significant inhibitory effects of chronic prenatal alcohol exposure on Jagged1 (Figure 7C), HES1 (Figure 7D), HIF-1α (Figure 7E), and FIH (Figure 7F) in the casein group, i.e., EC versus CC. Jagged1 and HIF-1α were also expressed at significantly lower levels in CS and ES than in CC. In contrast, the inhibitory effects of ethanol on HES1 and FIH in EC were not evident in the ES samples. No significant intergroup differences were observed with respect to ASPH (Figure 7A) or Notch1 (Figure 7B) mRNA transcripts.

### 3.7. Wnt Pathway mRNA Studies

Two-way ANOVA tests revealed significant dietary protein effects on Wnt5a, Fzd4, and Fzd6, as well as a statistical trend effect on Wnt5b (Table 4). Significant ethanol effects were detected for Wnt5a, Fzd6, EP300, and Axin2, and significant dietary protein × ethanol interactions were observed for Wnt5a, Wnt5b, Fzd6, Dixdc1, and Axin2. The corresponding graphs and post hoc tests revealed significantly lower expression levels of Wnt5a (Figure 8A), FZD6 (Figure 8D), Dixdc1 (Figure 8F), and Axin2 (Figure 8G) in EC, CS, and ES relative to CC controls. In addition, Wnt5b was significantly upregulated in ES relative to the other three groups (Figure 8B), whereas FZD4 was increased in CS and ES versus CC, reflecting a dietary soy stimulatory effect independent of ethanol exposure (Figure 8C). Chronic gestational ethanol exposure significantly reduced Fzd6 (Figure 8D), Dixdc (Figure 8F), and Axin2 in EC versus CC and EP300 in ES versus CS cerebella. EP300 was also trend-reduced in ES relative to CC (Figure 8E).

## 4. Discussion

This manuscript reports the effects of gestational dietary soy on postnatal cerebellar development and function in an experimental model of FASD. In previous reports utilizing moderate chronic ethanol feeding (26% caloric) of the pregnant dams, we demonstrated phenotypic craniofacial features of FASD in the late fetal/early postnatal period and linked those outcomes to impairments in placentation [46]. Importantly, ethanol-induced structural pathologies in the placenta were associated with significant abnormalities in the expression of molecules that mediate insulin and IGF signaling and ASPH, a critical downstream effector of placentation [19,46]. Remarkably, the co-administration of dietary soy to the pregnant dams largely abrogated the FASD features along with abnormalities in placentation [19,47]. The next goal was to determine if dietary soy restricted to the gestation period was sufficient to sustain or normalize neurodevelopment in the postnatal period in the FASD model. It is noteworthy that in previous studies of adult alcohol exposure models [48,49] and humans with alcohol use disorder [50,51,52], cessation of chronic alcohol exposure led to partial recovery of CNS pathology, suggesting that some aspects of alcohol-related brain degeneration may be reversible. These embedded questions are addressed in this experimental research report. Thus far, there are no published human studies in which dietary soy was used to ameliorate the effects of FASD, but clinical trials with choline supplementation have shown some promising effects. Choline is an abundant micronutrient in dietary soy. Another alternative approach recently published was the use of dietary docosahexaenoic acid and olive oil in pregnant rat dams that were chronically fed with high ethanol-containing diets, similar to the model herein [53]. The authors observed significant increases in the pups’ birth weights despite continued ethanol consumption.

The finding that weight gain trends in the pregnant dams and in the offspring were similar across the four experimental groups reaffirms that nutritional intake and ability to thrive were not compromised by prenatal alcohol exposures or differences in the dietary source of protein. However, prenatal dietary soy ultimately enhanced body growth in both control and gestational ethanol-exposed rats. On the other hand, the reduced mean brain weight in the EC group reflects the long-term effects of prenatal alcohol exposure on brain growth, as reported previously for experimental models [54] and humans [3,4,5]. The normalizing effect of dietary soy on brain weight in the P35 ES group is reassuring for soy’s significant positive impact on subsequent brain development. The normalizing effect of dietary soy on somatic growth corresponds with previous observations in fetal offspring using this model [19]. Mechanistically, dietary soy was shown to normalize placentation in chronic alcohol-exposed dams. Endocrine regulation of fetal growth by the production and metabolism of growth-regulating hormones, particularly IGFs, is an important placental function [55,56]. Similarly, IGFs have important growth-promoting effects on the developing brain [57]. Soy’s brain and body growth-promoting effects in development may have been due to stimulation of IGF-1 signaling pathways [58,59].

This study focused on cerebellar development and function to better understand how dietary soy limited to the prenatal period impacts a brain region that completes most of its robust development early (within the first two postnatal weeks in rodents) [60]. Purkinje cells are mainly formed prenatally [61]. Purkinje cell loss is an established feature of FASD [62]. The gestational alcohol exposure-associated loss of Purkinje cells was reduced by dietary soy. However, the overall differences between EC and ES were relatively modest, perhaps because postnatal cessation of alcohol exposure limited progressive neuronal loss. The findings with respect to the ethanol exposure effect on granule cells were more striking in the EC group, which exhibited pronounced hypoplasia corresponding with previous observations in experimental FASD [14]. In both the EC and ES groups, granule cell hypoplasia, marked by significantly reduced cellular densities, corresponded with impairments in motor function and learning that were not ameliorated by dietary soy administration to the dams during pregnancy.

An additional finding was the Luxol fast blue-staining pallor of cerebellar white matter in the EC brains, which was reduced by gestational dietary soy. Previous studies showed that cessation of chronic alcohol consumption partially reverses the histopathological and molecular/biochemical abnormalities in the cerebral white matter of adult experimental models of alcohol use disorder (AUD) [48]. Similarly, abstinence in humans with AUD at least partially restores white matter integrity along with cognitive function [51,52]. In our developmental model of FASD, cerebellar myelin integrity, marked by Luxol fast blue-staining pallor, and probable reductions in glia along with increased tissue vacuolation were partially prevented by prenatal dietary soy. White matter is an important target of alcohol-related brain injury across the lifespan [63]. The adverse effects of alcohol on white matter were shown to be mediated by impairments in insulin and IGF signaling through IRS-Akt-mTOR pathways [63]. Structural pathology in white matter contributes to functional impairments due to the loss of connections. In our rat model, compromised cerebellar white matter integrity likely contributed to the functional deficits observed by rotarod testing.

The rotarod tests showed that all experimental rat groups exhibited declines in performance with greater challenges related to increasing the speed of rod rotation. However, the EC and ES groups consistently performed worse, as was evidenced by their significantly shorter latencies to fall. Cerebellar granule cells are critical to cerebellar development and function, regulating the development of other cerebellar neuron types and establishing afferent connections [64]. Purkinje cells are the only output source from the cerebellar cortex to other brain regions that regulate motor function via inhibitory signaling to subcortical nuclei [64]. In contrast, granule cells are excitatory, and they receive afferent projections from outside the cerebellum. The elaborate network of cerebellar structural development with its synaptic connections is largely orchestrated during postnatal development and has pivotal roles in regulating motor function [64]. Therefore, the loss of cerebellar granule cells in the EC groups correlates well with the impairments in function. The persistence of prenatal alcohol-mediated loss of cerebellar granule cells, despite postnatal cessation of alcohol exposure and gestational dietary soy, reinforces the concept that normalizing cerebellar development and function in the postnatal period may require both cessation of alcohol exposure and continuous supplementation with dietary soy or related food/medicinal therapeutics.

The molecular and biochemical studies were designed to understand the signaling pathways and genes that were altered by prenatal alcohol exposure and dietary soy supplementation. The cerebellar samples were obtained 30 days after cessation of both exposures, contrasting with previous models in which the brains were harvested within the timeframe of ethanol and soy administration [19]. Therefore, the observed findings could have been mediated by the prolonged period of withdrawal with attendant spontaneous recovery from alcohol-mediated injury, negation of soy’s beneficial effects, or persistent impacts of prenatal alcohol and/or soy. The analysis focused on the insulin/IGF/IRS/Akt, Notch, and Wnt signaling pathways because previous studies showed their substantial vulnerabilities to alcohol exposure in the brain, including in development [45,65]. The significance of these findings is that FASD-related motor dysfunction is likely mediated by sustained inhibitory effects on multiple signaling pathways that crosstalk such that the impairments in each pathway adversely impact the others. However, it is also possible that by supporting the insulin/IGF/IRS/Akt pathway over the course of neurodevelopment via dietary soy or its phytonutrient constituents, Notch and Wnt signaling mechanisms would also be positively maintained. Importantly, future treatment strategies must consider the complexity of altered signaling mechanisms and attendant impairments in gene expression to more effectively treat or prevent long-term neurobehavioral effects of FASD.

In contrast to earlier findings in which chronic gestational exposure to alcohol was shown to inhibit insulin and IGF-1 receptor expression and signaling (tyrosine phosphorylation) through insulin receptor substrate molecules and downstream via activation of GSK-3β (increased protein expression and reduced S9 phosphorylation) [43], the findings herein revealed no significant inhibitory effects of ethanol on this network, as well as no increased activation of GSK-3β. Therefore, the specific ethanol effect on this pathway appeared to have been ameliorated or abolished by prolonged ethanol withdrawal. Instead, the main long-lasting effects were due to dietary soy, irrespective of ethanol exposure. The two- to four-fold increases in insulin and IGF-1 receptor expression may have been sufficient to enhance signaling overall, despite modest but statistically significant reductions in tyrosine phosphorylation. On the other hand, the soy-associated sharp reductions in IRS-1 protein, together with prominent increases in serine phosphorylation reflect long-term soy-mediated inhibition of signaling through IRS-1, which could account for the reduced levels of pS-Akt in both CS and ES cerebella. However, the optimum interpretation of these findings requires additional efforts to determine if IRS2 and IRS4 were similarly or differentially impacted by dietary soy. Increased signaling through the insulin and IGF-1 receptors mediated by higher levels of receptor expression could have served to support the growth and survival of cerebellar neurons and glial cells and myelin integrity, accounting for the modest improvements in motor function and better preservation of cellularity in the Purkinje and granule cell layers of ES versus EC cerebella.

In the brain, Notch pathways regulate neurodevelopment, modulating differentiation, migration, survival, and plasticity in neurons [66]. Notch signaling is mediated by interactions of Notch heterodimeric receptors with cell surface ligands such as Jagged expressed on nearby cells. Subsequent proteolytic cleavages release the Notch intracellular domain, resulting in its translocation to the nucleus where it binds to transcriptional regulators and ultimately leads to increased expression of hairy–enhancer of split (HES)-, HEY-, and HES-related protein gene families [66]. Major developmental outcomes of Notch signaling include neurogenesis, gliogenesis, neuronal migration, dendritic and synaptic plasticity, and behavioral functions linked to learning and memory [66,67]. ASPH was identified as a type 2 transmembrane protein with a C-terminal catalytic domain that hydroxylates aspartyl and asparaginyl residues in the EGF-like domains of Notch and Jagged [68,69,70,71]. Correspondingly, ASPH has demonstrated roles in neuronal migration, plasticity, growth, and survival, as well as glial functions via Notch pathway activation [23]. Hypoxia-inducible factor-1 alpha (HIF-1α) is a transcription factor that modulates cellular and tissue adaptive responses to hypoxia. HIF-1α has dual effects in inducing neuronal cell death in states of severe hypoxic or ischemia, but under milder circumstances, it can be neuroprotective and promote neuronal survival [72]. HIF-1α can mediate its effects by activating Notch via its physical interactions with the Notch intracellular domain [73]. Finally, the factor inhibiting HIF-1 (FIH), which like ASPH, has hydroxylase catalytic functions, mediates negatively regulated crosstalk between HIF and Notch by asparaginyl hydroxylation of the Notch ICD thereby inhibiting HIF activation of Notch [74,75]. High levels of FIH promote differentiation and inhibit immature functions such as neurogenesis [74]. In addition, HIF-1α’s inhibitory effects on energy metabolism and oxidative phosphorylation in mitochondria are inhibited by FIH [76].

The mRNA studies demonstrated significant inhibitory effects of ethanol on Notch pathway signaling through Jagged1, HES1, HIF-1α, and FIH in EC cerebellar samples. Definitive evidence of Notch pathway inhibition was marked by the significantly reduced expression of HES1. In previous studies of experimental FASD, Notch pathway signaling was also found to be reduced in the brain, but in the context of continued ethanol exposure [45]. The present work shows that ethanol-associated impairments in Notch signaling can persist for weeks after the exposures were discontinued and overlap with the critical period of postnatal cerebellar development. Previous studies established that inhibiting ASPH protein expression or hydroxylase activity, independent of its mRNA, impairs Notch signaling [77,78]. Furthermore, impaired cerebellar development in FASD models was found to be associated with reduced ASPH expression and catalytic activity [14] and corresponding inhibition of Notch [79]. In this model, although ASPH mRNA expression was not altered by ethanol, the significantly reduced expression of HES1 corresponds with Notch pathway inhibition, possibly mediated by the persistent inhibition of ASPH activity. Additional potential mediators of ethanol-impaired Notch activation include reduced Jagged1 ligands and HIF-1α expression. The reduced FIH expression in EC versus CC cerebella may reflect a compensatory protective response against further inhibiting Notch vis à vis the significantly reduced HIF-1α.

Dietary soy’s normalization of HES1 in ES cerebella suggests that its administration in the prenatal period was sufficient to restore Notch signaling during postnatal cerebellar development. The positive effects of dietary soy were evidenced by improvements in the anatomical structure of the cerebellum and the normalization of brain weight. However, the increased HES1 expression was not accompanied by parallel increases in Jagged1 or HIF-1α. We postulate that since oxidative stress and inflammation activate Notch networks [80], soy’s potent antioxidant and anti-inflammatory effects [34,81,82,83] account for the seemingly paradoxical enhancement of HES1 expression with inhibition of HIF-1α. The soy-mediated inhibition of HIF-1α may have been beneficial in helping to preserve neuronal survival by reducing attendant oxidative stress, inflammation, and mitochondrial dysfunction [84,85,86], and accounts for the corresponding better preservation of cerebellar development despite chronic prenatal alcohol exposure. However, it is critical to appreciate that Notch and HIF-1α signaling effects can positively or negatively impact cellular functions. Therefore, additional mechanistic studies are needed to dissect the physiological and pathophysiological responses linked to the independent and combined effects of ethanol and dietary soy exposure and withdrawal in the period of postnatal brain development.

Wingless-related integration site (Wnt) genes encode ligands with diverse biological functions and are critical for development, including in the brain [25]. Wnt/β-catenin signaling has important roles in neurodevelopment and is adversely impacted by chronic alcohol exposures [45]. Our studies were focused on Wnt5a, which supports neurite outgrowth and synapse formation in immature neurons [87,88] and is a critical regulator of neurogenesis during cerebellar development [89], and Wnt5b, which is related to Wnt5a and needed for cell migration, proliferation, and differentiation, but signals through non-canonical Wnt which is β-catenin-independent. Wnt5b has critical roles in cerebellar development including cerebellar granule neuron generation [90]. The Frizzled 4 (Fzd4) receptor regulates canonical pathway molecules expressed in cerebellar Purkinje cells but also plays roles in the blood–retinal and blood–brain barrier functions. Fzd6, a noncanonical pathway receptor critical for Wnt signaling via ligand binding, mediates important functions during development, including synapse formation, synaptic plasticity, and neuronal circuitry needed for diverse CNS functions [91]. Fzd6 also inhibits the canonical pathway, reducing b-catenin target gene expression via TCF/LEF transcription factor phosphorylation [92]. The histone acetyltransferase EP300 interacts with erythropoietin receptors and supports neuroprotective and neuro-regenerative functions [93]. In addition, EP300 suppresses hippocampal and canonical Wnt pathways [94]. Disheveled-Axin domain containing-1 (Dixcd1) is a critical canonical pathway regulator of neurodevelopment with roles in neuronal migration [95]. Axin2 is expressed in immature brain oligodendrocyte progenitor cells in white matter and has a critical role in remyelination [96].

The EC-associated significant reduction in mRNA expression of Wnt5a, Fzd6, Dixdc1, and Axin2 indicated that chronic prenatal alcohol exposures have long-lasting sustained inhibitory effects on both canonical (Wnt5a, Dixcd1, Axin2) and non-canonical (Fzd6) Wnt pathways, which may account for the persistent impairments in cerebellar neurodevelopment and function. The partial restoration or normalization of cerebellar development in the ES samples was associated with the increased expression of signaling molecules that mediate canonical Wnt/β-catenin (Fzd4 and Dixdc1) or non-canonical (Wnt5b). The positive stimulatory effects of prenatal dietary soy in the ES group may have aided in supporting cerebellar cortical neurogenesis, neuronal plasticity, Purkinje cell functions, and white matter development. However, the failure to restore cerebellar motor function could have been mediated by incomplete or un-sustained effects of dietary soy due to a lack of exposure during postnatal cerebellar development and maturation. In addition, relatively reduced EP300 in ES versus CS samples may have contributed to incomplete recovery given its critical roles in neuroprotective and neuro-regenerative erythropoietin receptors [93].

Limitations of the research and interpretation of results include the following: (1) the lack of data concerning the longer-term follow-up to determine the degrees to which the motor deficits and cerebellar pathology normalize with further maturation; (2) insufficient study design power to fully evaluate sex as a biological variable; (3) the lack of specific experimental data that directly address the mechanisms mediating the cerebellar pathology and motor impairments; (4) inability to determine if the FASD effects in humans are caused by ethanol’s inhibitory effects on signal transduction in the immature brain, or the combined effects of poor nutrition and ethanol neurotoxicity; and (5) uncertainty about the mechanisms of dietary soy’s neuroprotection. The first two limitations could be addressed in future studies by conducting a timepoint analysis of cerebellar structure and function through early adulthood and expanding the number of animals to include balanced larger numbers of both males and females. Mechanistic experiments could be conducted by treating control and ethanol-exposed cerebellar slice cultures with a cell-permeable Wnt agonist such as BML-284 or a Notch activator such as Yhhu 3792. Alternatively, chemical inhibitors of Wnt (MSAB selective inhibitor of Wnt/β-catenin) or Notch (γ-secretase inhibitor) could be used to determine the degree to which they mimic the adverse effects of ethanol on cerebellar neuron and glial cell functions. The experiments herein were conducted with isocaloric diets with adequate nutrients, including proteins, yet poor nutrition may exacerbate the adverse effects of prenatal alcohol exposure. Although this hypothesis could be tested experimentally, in the clinical realm, the question could be addressed by evaluating outcomes in nutritionally supported populations versus historical controls. In addition, the supply of dietary soy, besides providing important phytonutrients, is an excellent source of protein that could help ameliorate the additive effects of poor nutrition vis à vis chronic gestational ethanol exposure. Mechanistically, due to their insulin-sensitizing and antioxidant effects, the isoflavones in dietary soy could have had a major positive impact on neurodevelopment in the prenatal ethanol exposure model. Furthermore, the high content of choline in soy (https://www.healthline.com/nutrition/foods-with-choline accessed on 24 February 2025), may also have had significant rescue effects in the FASD model [97,98]. Further studies should determine the degrees to which prenatal alcohol exposure effects can be abrogated by pair-feeding the dams with natural versus alcohol-stripped soy isolate or supplementing the casein liquid diets with different concentrations of choline.

## 5. Conclusions

This study examined the effects of maternal dietary soy on postnatal cerebellar development in an FASD Long Evans rat model. Alcohol exposure and soy administration were limited to the period of gestation.

Dietary soy prevented alcohol-related reductions in brain weight and significantly increased cerebellar granule neuron density in ethanol-exposed offspring.

Despite improvements in cerebellar structure, motor performance was equally impaired in the ethanol-exposed offspring of dams fed with soy or casein (control) as the dietary protein source.

Postnatal cessation of alcohol exposure abolished the impairments in insulin/IGF-1/IRS1 pathway signaling that were previously associated with ongoing ethanol exposures.

Gestational dietary soy’s abrogation of ethanol’s persistent inhibitory effects on Notch (HES-1, FIH) and Wnt (Fzd4) and up-regulation of Wnt5b, insulin receptor, and IGF-1 receptor could account for the associated significant improvements in cerebellar structure.

Optimum prevention of FASD’s effects on neurodevelopment and function will likely require dietary soy interventions throughout gestation and the critical periods of postnatal development, even when alcohol exposure has ceased.

## Figures and Tables

**Table 1:** Maternal and offspring weights—ANOVA tests.

Variable	Diet F-Ratio	*p*-Value	Ethanol F-Ratio	*p*-Value	Interaction F-Ratio	*p*-Value
**Dam’s Weight GD0**	3.363	0.175	2.496	0.153	1.698	0.229
**Dam’s Weight-GD21/P0**	0.075	0.791	1.775	0.327	1.088	0.328
**Offspring P0 Birth Weight**	0.129	0.722	1.075	0.302	0.676	0.417
**Offspring P35 Body Weight**	**14.41**	**0.0006**	**6.119**	**0.0185**	0.358	0.554
**Offspring P35 Brain Weight**	**7.491**	**0.0096**	*3.229*	*0.08*	**5.413**	**0.0257**

Results of two-way ANOVA tests to examine the effects of diet (soy versus casein), ethanol, and diet × ethanol interactions on maternal weight (at delivery), birth weight in the offspring, body weight of the offspring at P35 (experimental endpoint), and brain weight of the offspring at P35. The results were analyzed with Graphpad Prism 10.2 software. The calculated F-ratios and *p*-values are displayed [DF: Maternal Weight GD0 and GD21 (DFn = 1, DFd = 12); birth weight, P35 body weight, and P35 brain weight (DFn = 1, DFd = 118)]. Significant differences are highlighted with bold fonts. Statistical trendwise differences (0.05 < *p* < 0.10) are italicized N = 25–41 offspring per group. See graphs and post hoc test results in Figure 1.

## Data Availability

The data underlying this article will be shared upon reasonable request to the corresponding author.

## Supplementary Materials

The following supporting information can be downloaded at: https://www.mdpi.com/article/10.3390/nu17050812/s1, Table S1: Commercial Multiplex ELISA Platforms. Table S2: Reagents for TaqMan mRNA Expression Studies. Table S3: Characteristics of Diets Used in. Table S4: Effects of Dietary Protein Source and Ethanol on Body and Brain Weights. Table S5: Analysis of Sex Effects; Mixed-Model ANOVA with the Šídák’s Multiple Comparisons Test.

## References

[1] Nayak R.B., Murthy P. (2008). Fetal alcohol spectrum disorder. Indian Pediatr..

[2] Osborn J.A., Harris S.R., Weinberg J. (1993). Fetal alcohol syndrome: Review of the literature with implications for physical therapists. Phys. Ther..

[3] Moore E.M., Xia Y. (2021). Neurodevelopmental Trajectories Following Prenatal Alcohol Exposure. Front. Hum. Neurosci..

[4] Riley E.P., Infante M.A., Warren K.R. (2011). Fetal alcohol spectrum disorders: An overview. Neuropsychol. Rev..

[5] Riley E.P., McGee C.L. (2005). Fetal alcohol spectrum disorders: An overview with emphasis on changes in brain and behavior. Exp. Biol. Med..

[6] Sullivan E.V., Moore E.M., Lane B., Pohl K.M., Riley E.P., Pfefferbaum A. (2020). Graded Cerebellar Lobular Volume Deficits in Adolescents and Young Adults with Fetal Alcohol Spectrum Disorders (FASD). Cereb. Cortex.

[7] Chanraud S., Martelli C., Delain F., Kostogianni N., Douaud G., Aubin H.J., Reynaud M., Martinot J.L. (2007). Brain morphometry and cognitive performance in detoxified alcohol-dependents with preserved psychosocial functioning. Neuropsychopharmacol. Off. Publ. Am. Coll. Neuropsychopharmacol..

[8] Bookstein F.L., Streissguth A.P., Connor P.D., Sampson P.D. (2006). Damage to the human cerebellum from prenatal alcohol exposure: The anatomy of a simple biometrical explanation. Anat. Rec. B New Anat..

[9] Norman A.L., Crocker N., Mattson S.N., Riley E.P. (2009). Neuroimaging and fetal alcohol spectrum disorders. Dev. Disabil. Res. Rev..

[10] Dorrie N., Focker M., Freunscht I., Hebebrand J. (2014). Fetal alcohol spectrum disorders. Eur. Child Adolesc. Psychiatry.

[11] Sawant O.B., Lunde E.R., Washburn S.E., Chen W.J., Goodlett C.R., Cudd T.A. (2013). Different patterns of regional Purkinje cell loss in the cerebellar vermis as a function of the timing of prenatal ethanol exposure in an ovine model. Neurotoxicol. Teratol..

[12] Cealie M.Y., Douglas J.C., Swan H.K., Vonkaenel E.D., McCall M.N., Drew P.D., Majewska A.K. (2024). Developmental Ethanol Exposure Impacts Purkinje Cells but Not Microglia in the Young Adult Cerebellum. Cells.

[13] Gonzalez-Burgos I., Alejandre-Gomez M. (2005). Cerebellar granule cell and Bergmann glial cell maturation in the rat is disrupted by pre- and post-natal exposure to moderate levels of ethanol. Int. J. Dev. Neurosci..

[14] de la Monte S.M., Tong M., Carlson R.I., Carter J.J., Longato L., Silbermann E., Wands J.R. (2009). Ethanol inhibition of aspartyl-asparaginyl-beta-hydroxylase in fetal alcohol spectrum disorder: Potential link to the impairments in central nervous system neuronal migration. Alcohol.

[15] Niedzwiedz-Massey V.M., Douglas J.C., Rafferty T., Kane C.J.M., Drew P.D. (2021). Ethanol effects on cerebellar myelination in a postnatal mouse model of fetal alcohol spectrum disorders. Alcohol.

[16] Adamo M., Raizada M.K., LeRoith D. (1989). Insulin and insulin-like growth factor receptors in the nervous system. Mol. Neurobiol..

[17] Torres-Aleman I., Pons S., Arevalo M.A. (1994). The insulin-like growth factor I system in the rat cerebellum: Developmental regulation and role in neuronal survival and differentiation. J. Neurosci. Res..

[18] de la Monte S.M., Tong M., Bowling N., Moskal P. (2011). si-RNA inhibition of brain insulin or insulin-like growth factor receptors causes developmental cerebellar abnormalities: Relevance to fetal alcohol spectrum disorder. Mol. Brain.

[19] Qi W., Gundogan F., Gilligan J., Monte S. (2023). Dietary soy prevents fetal demise, intrauterine growth restriction, craniofacial dysmorphic features, and impairments in placentation linked to gestational alcohol exposure: Pivotal role of insulin and insulin-like growth factor signaling networks. Alcohol.

[20] Lawton M., Tong M., Gundogan F., Wands J.R., de la Monte S.M. (2010). Aspartyl-(asparaginyl) beta-hydroxylase, hypoxia-inducible factor-alpha and Notch cross-talk in regulating neuronal motility. Oxid. Med. Cell Longev..

[21] Gundogan F., Elwood G., Greco D., Rubin L.P., Pinar H., Carlson R.I., Wands J.R., de la Monte S.M. (2007). Role of aspartyl-(asparaginyl) beta-hydroxylase in placental implantation: Relevance to early pregnancy loss. Hum. Pathol..

[22] Cantarini M.C., de la Monte S.M., Pang M., Tong M., D’Errico A., Trevisani F., Wands J.R. (2006). Aspartyl-asparagyl beta hydroxylase over-expression in human hepatoma is linked to activation of insulin-like growth factor and notch signaling mechanisms. Hepatology.

[23] Tong M., Gao J.S., Borgas D., de la Monte S.M. (2013). Phosphorylation Modulates Aspartyl-(Asparaginyl)-beta Hydroxylase Protein Expression, Catalytic Activity and Migration in Human Immature Neuronal Cerebellar Cells. Cell Biol..

[24] Dietrich B., Haider S., Meinhardt G., Pollheimer J., Knofler M. (2022). WNT and NOTCH signaling in human trophoblast development and differentiation. Cell Mol. Life Sci..

[25] Mehta S., Hingole S., Chaudhary V. (2021). The Emerging Mechanisms of Wnt Secretion and Signaling in Development. Front. Cell Dev. Biol..

[26] Wong S.K., Mohamad N.V., Jayusman P.A., Ibrahim N. (2023). A Review on the Crosstalk between Insulin and Wnt/beta-Catenin Signalling for Bone Health. Int. J. Mol. Sci..

[27] Palsgaard J., Emanuelli B., Winnay J.N., Sumara G., Karsenty G., Kahn C.R. (2012). Cross-talk between insulin and Wnt signaling in preadipocytes: Role of Wnt co-receptor low density lipoprotein receptor-related protein-5 (LRP5). J. Biol. Chem..

[28] Collu G.M., Hidalgo-Sastre A., Brennan K. (2014). Wnt-Notch signalling crosstalk in development and disease. Cell Mol. Life Sci..

[29] Acar A., Hidalgo-Sastre A., Leverentz M.K., Mills C.G., Woodcock S., Baron M., Collu G.M., Brennan K. (2021). Inhibition of Wnt signalling by Notch via two distinct mechanisms. Sci. Rep..

[30] Lange S., Probst C., Gmel G., Rehm J., Burd L., Popova S. (2017). Global Prevalence of Fetal Alcohol Spectrum Disorder Among Children and Youth: A Systematic Review and Meta-analysis. JAMA Pediatr..

[31] Tong M., Dominguez C., Didsbury J., de la Monte S.M. (2016). Targeting Alzheimer’s Disease Neuro-Metabolic Dysfunction with a Small Molecule Nuclear Receptor Agonist (T3D-959) Reverses Disease Pathologies. J. Alzheimers Dis. Park..

[32] Xu B., Xing A., Li S. (2022). The forgotten type 2 diabetes mellitus medicine: Rosiglitazone. Diabetol. Int..

[33] Zhang J., Liu X., Xie X.B., Cheng X.C., Wang R.L. (2016). Multitargeted bioactive ligands for PPARs discovered in the last decade. Chem. Biol. Drug Des..

[34] Hassan S.M., El-Shemy H.A. (2013). Soybean, Nutrition and Health. Soybean-Bio-Active Compounds.

[35] Tong M., Ziplow J.L., Mark P., de la Monte S.M. (2022). Dietary Soy Prevents Alcohol-Mediated Neurocognitive Dysfunction and Associated Impairments in Brain Insulin Pathway Signaling in an Adolescent Rat Model. Biomolecules.

[36] Westerhuis J.A.W., Dudink J., Wijnands B., De Zeeuw C.I., Canto C.B. (2024). Impact of Intrauterine Insults on Fetal and Postnatal Cerebellar Development in Humans and Rodents. Cells.

[37] Gundogan F., Tong M., Monte S.M.d.l. (2024). Association between dietary soy prevention of fetal alcohol spectrum disorder and normalization of placental insulin and insulin-like growth factor signaling networks and downstream effector molecule expression. Gene Protein Dis..

[38] Gundogan F., Elwood G., Longato L., Tong M., Feijoo A., Carlson R.I., Wands J.R., de la Monte S.M. (2008). Impaired placentation in fetal alcohol syndrome. Placenta.

[39] Genovese M.I., Barbosa A.C., Pinto Mda S., Lajolo F.M. (2007). Commercial soy protein ingredients as isoflavone sources for functional foods. Plant Foods Hum. Nutr..

[40] Gianazza E., Eberini I., Arnoldi A., Wait R., Sirtori C.R. (2003). A proteomic investigation of isolated soy proteins with variable effects in experimental and clinical studies. J. Nutr..

[41] Jimenez J.A., Zylka M.J. (2021). Controlling litter effects to enhance rigor and reproducibility with rodent models of neurodevelopmental disorders. J. Neurodev. Disord..

[42] Lazic S.E., Essioux L. (2013). Improving basic and translational science by accounting for litter-to-litter variation in animal models. BMC Neurosci..

[43] Ewenczyk A., Ziplow J., Tong M., Le T., de la Monte S.M. (2012). Sustained Impairments in Brain Insulin/IGF Signaling in Adolescent Rats Subjected to Binge Alcohol Exposures during Development. J. Clin. Exp. Pathol..

[44] Woo J.R., Bae S.H., Wales T.E., Engen J.R., Lee J., Jang H., Park S. (2024). The serine phosphorylations in the IRS-1 PIR domain abrogate IRS-1 and IR interaction. Proc. Natl. Acad. Sci. USA.

[45] Tong M., Ziplow J., Chen W.C., Nguyen Q.G., Kim C., de la Monte S.M. (2013). Motor Function Deficits Following Chronic Prenatal Ethanol Exposure are Linked to Impairments in Insulin/IGF, Notch and Wnt Signaling in the Cerebellum. J. Diabetes Metab..

[46] Gundogan F., Gilligan J., Qi W., Chen E., Naram R., de la Monte S.M. (2015). Dose effect of gestational ethanol exposure on placentation and fetal growth. Placenta.

[47] Gundogan F., Qi W., Gilligan J., de la Monte S. (2013). Effects of dietary soy on ethanol-impaired placentation and fetal growth. Placenta.

[48] Yalcin E.B., Tong M., de la Monte S.M. (2018). Altered Oligodendroglial and Neuroglial Gene Expression in Adult Rat Cerebral White Matter Following Short- and Long-Term Ethanol Exposures and Brief Abstinence. J. Drug Alc. Res..

[49] Crews F.T., Nixon K. (2009). Mechanisms of neurodegeneration and regeneration in alcoholism. Alcohol Alcohol..

[50] Meyerhoff D.J., Bloomer C., Schuff N., Ezekiel F., Norman D., Clark W., Weiner M.W., Fein G. (1999). Cortical metabolite alterations in abstinent cocaine and cocaine/alcohol-dependent subjects: Proton magnetic resonance spectroscopic imaging. Addict. Biol..

[51] Monnig M.A., Tonigan J.S., Yeo R.A., Thoma R.J., McCrady B.S. (2013). White matter volume in alcohol use disorders: A meta-analysis. Addict. Biol..

[52] Pfefferbaum A., Adalsteinsson E., Sullivan E.V. (2006). Dysmorphology and microstructural degradation of the corpus callosum: Interaction of age and alcoholism. Neurobiol. Aging.

[53] Yadav D., Ostrea E.M., Cheng C.T., Kisseih E., Maddipati K.R., Thomas R.L. (2024). Effect of docosahexaenoic acid and olive oil supplementation on pup weight in alcohol-exposed pregnant rats. Front. Pediatr..

[54] Green C.R., Kobus S.M., Ji Y., Bennett B.M., Reynolds J.N., Brien J.F. (2005). Chronic prenatal ethanol exposure increases apoptosis in the hippocampus of the term fetal guinea pig. Neurotoxicol. Teratol..

[55] Murphy V.E., Smith R., Giles W.B., Clifton V.L. (2006). Endocrine regulation of human fetal growth: The role of the mother, placenta, and fetus. Endocr. Rev..

[56] Forbes K., Westwood M. (2010). Maternal growth factor regulation of human placental development and fetal growth. J. Endocrinol..

[57] McDonald T.J., Nijland M.J., Nathanielsz P.W. (2007). The insulin-like growth factor system and the fetal brain: Effects of poor maternal nutrition. Rev. Endocr. Metab. Disord..

[58] Khalil D.A., Lucas E.A., Juma S., Smith B.J., Payton M.E., Arjmandi B.H. (2002). Soy protein supplementation increases serum insulin-like growth factor-I in young and old men but does not affect markers of bone metabolism. J. Nutr..

[59] Gao Q.G., Xie J.X., Wong M.S., Chen W.F. (2012). IGF-I receptor signaling pathway is involved in the neuroprotective effect of genistein in the neuroblastoma SK-N-SH cells. Eur. J. Pharmacol..

[60] Rocamora N., Garcia-Ladona F.J., Palacios J.M., Mengod G. (1993). Differential expression of brain-derived neurotrophic factor, neurotrophin-3, and low-affinity nerve growth factor receptor during the postnatal development of the rat cerebellar system. Brain Res. Mol. Brain Res..

[61] Vinters H.V., Gatti R.A., Rakic P. (1985). Sequence of cellular events in cerebellar ontogeny relevant to expression of neuronal abnormalities in ataxia-telangiectasia. Kroc Found. Ser..

[62] West J.R., Parnell S.E., Chen W.J., Cudd T.A. (2001). Alcohol-mediated Purkinje cell loss in the absence of hypoxemia during the third trimester in an ovine model system. Alcohol. Clin. Exp. Res..

[63] de La Monte S.M., Sutherland G.T. Dual Stages of Alcohol-Related Cerebral White Matter Degeneration Reviewed: Early-Stage Stress/Neuroinflammation Versus Late-Stage Impaired Insulin/IGF Signaling Through Akt-mTOR—Review. ASN Neuro.

[64] Kim M., Jun S., Park H., Tanaka-Yamamoto K., Yamamoto Y. (2023). Regulation of cerebellar network development by granule cells and their molecules. Front. Mol. Neurosci..

[65] de la Monte S.M., Yeon J.E., Tong M., Longato L., Chaudhry R., Pang M.Y., Duan K., Wands J.R. (2008). Insulin resistance in experimental alcohol-induced liver disease. J. Gastroenterol. Hepatol..

[66] Lasky J.L., Wu H. (2005). Notch signaling, brain development, and human disease. Pediatr. Res..

[67] Ables J.L., Breunig J.J., Eisch A.J., Rakic P. (2011). Not(ch) just development: Notch signalling in the adult brain. Nat. Rev. Neurosci..

[68] Dinchuk J.E., Henderson N.L., Burn T.C., Huber R., Ho S.P., Link J., O’Neil K.T., Focht R.J., Scully M.S., Hollis J.M. (2000). Aspartyl beta -hydroxylase (Asph) and an evolutionarily conserved isoform of Asph missing the catalytic domain share exons with junctin. J. Biol. Chem..

[69] Ince N., de la Monte S.M., Wands J.R. (2000). Overexpression of human aspartyl (asparaginyl) beta-hydroxylase is associated with malignant transformation. Cancer Res..

[70] Jia S., VanDusen W.J., Diehl R.E., Kohl N.E., Dixon R.A., Elliston K.O., Stern A.M., Friedman P.A. (1992). cDNA cloning and expression of bovine aspartyl (asparaginyl) beta-hydroxylase. J. Biol. Chem..

[71] McGinnis K., Ku G.M., VanDusen W.J., Fu J., Garsky V., Stern A.M., Friedman P.A. (1996). Site-directed mutagenesis of residues in a conserved region of bovine aspartyl (asparaginyl) beta-hydroxylase: Evidence that histidine 675 has a role in binding Fe^2+^. Biochemistry.

[72] Cheng Y.L., Park J.S., Manzanero S., Choi Y., Baik S.H., Okun E., Gelderblom M., Fann D.Y., Magnus T., Launikonis B.S. (2014). Evidence that collaboration between HIF-1alpha and Notch-1 promotes neuronal cell death in ischemic stroke. Neurobiol. Dis..

[73] Li Y., Wu L., Yu M., Yang F., Wu B., Lu S., Tu M., Xu H. (2018). HIF-1alpha is Critical for the Activation of Notch Signaling in Neurogenesis During Acute Epilepsy. Neuroscience.

[74] Zheng X., Linke S., Dias J.M., Zheng X., Gradin K., Wallis T.P., Hamilton B.R., Gustafsson M., Ruas J.L., Wilkins S. (2008). Interaction with factor inhibiting HIF-1 defines an additional mode of cross-coupling between the Notch and hypoxia signaling pathways. Proc. Natl. Acad. Sci. USA.

[75] Coleman M.L., McDonough M.A., Hewitson K.S., Coles C., Mecinovic J., Edelmann M., Cook K.M., Cockman M.E., Lancaster D.E., Kessler B.M. (2007). Asparaginyl hydroxylation of the Notch ankyrin repeat domain by factor inhibiting hypoxia-inducible factor. J. Biol. Chem..

[76] Weidemann A., Johnson R.S. (2008). Biology of HIF-1alpha. Cell Death Differ..

[77] Gundogan F., Bedoya A., Gilligan J., Lau E., Mark P., De Paepe M.E., de la Monte S.M. (2011). siRNA inhibition of aspartyl-asparaginyl beta-hydroxylase expression impairs cell motility, Notch signaling, and fetal growth. Pathol. Res. Pr..

[78] Aihara A., Huang C.K., Olsen M.J., Lin Q., Chung W., Tang Q., Dong X., Wands J.R. (2014). A cell-surface beta-hydroxylase is a biomarker and therapeutic target for hepatocellular carcinoma. Hepatology.

[79] Silbermann E., Moskal P., Bowling N., Tong M., de la Monte S.M. (2010). Role of aspartyl-(asparaginyl)-beta-hydroxylase mediated notch signaling in cerebellar development and function. Behav. Brain Funct..

[80] Boopathy A.V., Pendergrass K.D., Che P.L., Yoon Y.S., Davis M.E. (2013). Oxidative stress-induced Notch1 signaling promotes cardiogenic gene expression in mesenchymal stem cells. Stem Cell Res. Ther..

[81] Clark J.L., Taylor C.G., Zahradka P. (2018). Rebelling against the (Insulin) Resistance: A Review of the Proposed Insulin-Sensitizing Actions of Soybeans, Chickpeas, and Their Bioactive Compounds. Nutrients.

[82] Tovar A.R., Torre-Villalvazo I., Ochoa M., Elias A.L., Ortiz V., Aguilar-Salinas C.A., Torres N. (2005). Soy protein reduces hepatic lipotoxicity in hyperinsulinemic obese Zucker fa/fa rats. J. Lipid Res..

[83] Wagner J.D., Zhang L., Shadoan M.K., Kavanagh K., Chen H., Tresnasari K., Kaplan J.R., Adams M.R. (2008). Effects of soy protein and isoflavones on insulin resistance and adiponectin in male monkeys. Metabolism.

[84] Halterman M.W., Miller C.C., Federoff H.J. (1999). Hypoxia-Inducible Factor-1α Mediates Hypoxia-Induced Delayed Neuronal Death That Involves p53. J. Neurosci..

[85] Tang Y.Y., Wang D.C., Wang Y.Q., Huang A.F., Xu W.D. (2022). Emerging role of hypoxia-inducible factor-1alpha in inflammatory autoimmune diseases: A comprehensive review. Front. Immunol..

[86] Mialet-Perez J., Belaidi E. (2024). Interplay between hypoxia inducible Factor-1 and mitochondria in cardiac diseases. Free Radic. Biol. Med..

[87] Halleskog C., Mulder J., Dahlstrom J., Mackie K., Hortobagyi T., Tanila H., Kumar Puli L., Farber K., Harkany T., Schulte G. (2011). WNT signaling in activated microglia is proinflammatory. Glia.

[88] Chen C.M., Orefice L.L., Chiu S.L., LeGates T.A., Hattar S., Huganir R.L., Zhao H., Xu B., Kuruvilla R. (2017). Wnt5a is essential for hippocampal dendritic maintenance and spatial learning and memory in adult mice. Proc. Natl. Acad. Sci. USA.

[89] Subashini C., Dhanesh S.B., Chen C.M., Riya P.A., Meera V., Divya T.S., Kuruvilla R., Buttler K., James J. (2017). Wnt5a is a crucial regulator of neurogenesis during cerebellum development. Sci. Rep..

[90] Suthon S., Perkins R.S., Bryja V., Miranda-Carboni G.A., Krum S.A. (2021). WNT5B in Physiology and Disease. Front. Cell Dev. Biol..

[91] Pascual-Vargas P., Salinas P.C. (2021). A Role for Frizzled and Their Post-Translational Modifications in the Mammalian Central Nervous System. Front. Cell Dev. Biol..

[92] Corda G., Sala A. (2017). Non-canonical WNT/PCP signalling in cancer: Fzd6 takes centre stage. Oncogenesis.

[93] Ott C., Martens H., Hassouna I., Oliveira B., Erck C., Zafeiriou M.P., Peteri U.K., Hesse D., Gerhart S., Altas B. (2015). Widespread Expression of Erythropoietin Receptor in Brain and Its Induction by Injury. Mol. Med..

[94] Rouhi L., Fan S., Cheedipudi S.M., Braza-Boils A., Molina M.S., Yao Y., Robertson M.J., Coarfa C., Gimeno J.R., Molina P. (2022). The EP300/TP53 pathway, a suppressor of the Hippo and canonical WNT pathways, is activated in human hearts with arrhythmogenic cardiomyopathy in the absence of overt heart failure. Cardiovasc. Res..

[95] Singh K.K., Ge X., Mao Y., Drane L., Meletis K., Samuels B.A., Tsai L.H. (2010). Dixdc1 is a critical regulator of DISC1 and embryonic cortical development. Neuron.

[96] Fancy S.P., Harrington E.P., Yuen T.J., Silbereis J.C., Zhao C., Baranzini S.E., Bruce C.C., Otero J.J., Huang E.J., Nusse R. (2011). Axin2 as regulatory and therapeutic target in newborn brain injury and remyelination. Nat. Neurosci..

[97] Thomas J.D., Idrus N.M., Monk B.R., Dominguez H.D. (2010). Prenatal choline supplementation mitigates behavioral alterations associated with prenatal alcohol exposure in rats. Birth Defects Res. Part A Clin. Mol. Teratol..

[98] Wozniak J.R., Fink B.A., Fuglestad A.J., Eckerle J.K., Boys C.J., Sandness K.E., Radke J.P., Miller N.C., Lindgren C., Brearley A.M. (2020). Four-year follow-up of a randomized controlled trial of choline for neurodevelopment in fetal alcohol spectrum disorder. J. Neurodev. Disord..

