# Supplementary material for: Impact of Prenatal Dietary Soy on Cerebellar Neurodevelopment and Function in Experimental Fetal Alcohol Spectrum Disorder
Source: Nutrients. 2025 Feb 26;17(5):812. doi: 10.3390/nu17050812 (PMC11901751; doi:10.3390/nu17050812)
Supplement: Supplementary file 1 [file nutrients-17-00812-s001.zip › nutrients-3405917-SM.pdf]

**Supplementary Table S1: Commercial Multiplex ELISA Platforms**

| Antibody Total Protein Targets | Antibody Phospho-Protein Targets |
|---|---|
| IR (Insulin Receptor) | IR (Tyr1162/1163) |
| IGF-1R (Insulin-Like Growth Factor Receptor, Type 1) | IGF-1R (Tyr1135/1136) |
| IRS1 (Insulin Receptor Substrate, Type 1) | IRS1 (Ser636) |
| Akt (Protein Kinase B) | Akt (Ser473) |
| GSK-3β (Glycogen Synthase Kinase 3β) | GSK-3β (Ser9) |

Invitrogen (Carlsbad, CA, USA) Total AKT (LHO0002M) and Phospho-AKT (LHO0001M) rat Magnetic Bead Multiplex ELISA Kits were used to measure ethanol and dietary soy effects on this signaling pathway. The left column lists the standard molecule abbreviations along with the full names in parenthesis. The right column indicates the specific phosphorylation sites (in parenthesis) corresponding to the target molecules assayed in the phospho-AKT ELISA kit.

Supplementary Table S2: Reagents for TaqMan mRNA Expression Studies

| Vendor | Catalog # | Description | Probe Target |
|---|---|---|---|
| Roche Applied Biosystems | 4369510 | TagMan gene expression master mix | |
| Roche Applied Science | 05 046 203 001 | Rat ACTB gene assay | β-Actin |
| Roche Applied Science | 04 686 993 001 | UPL probe #25 | ASPH |
| Roche Applied Science | 04 693 566 001 | UPL probe #122 | Notch1 |
| Roche Applied Science | 04 688 643 001 | UPL probe #65 | Jagged1 |
| Roche Applied Science | 04 689 011 001 | UPL probe #78 | HES1 |
| Roche Applied Science | 04 685 148 001 | UPL probe #15 | HIF-1α |
| Roche Applied Science | 04 685 148 001 | UPL probe #15 | FIH |
| Roche Applied Science | 04 692 098 001 | UPL probe #92 | Wnt5a |
| Roche Applied Science | 04 689 011 001 | UPL probe #78 | Wnt5b |
| Roche Applied Science | 04 688 651 001 | UPL probe #66 | Fzd6 |
| Roche Applied Science | 04 688 015 001 | UPL probe #42 | Fzd4 |
| Roche Applied Science | 04 688 066 001 | UPL probe #46 | Dixdc1 |
| Roche Applied Science | 04 688 619 001 | UPL probe #62 | Ep300 |

Roche Applied Biosystems Taqman reagents and probes were used to measure mRNA expression in rat frontal lobe tissue by the probe hydrolysis method, with β-Actin serving as a negative control.

Supplementary Table S3: Characteristics of Diets Used in

Experiments 3A: Compositions of Rodent Liquid and Chow Diets

| Ingredients | Standard Liquid Diets (F1259SP and F1258SP) | Experimental Liquid F5736SP and F5737SP) | Chow (F3197 AIN-93) |
|---|---|---|---|
| Protein | Casein | Soy Isolate | Casein |
| Carbohydrate | Maltodextrin, Corn syrup solids | Maltodextrin, Corn syrup solids | Maltodextrin, Sucrose |
| Fats | Olive Oil, Corn Oil, Safflower Oil | Olive Oil, Corn Oil, Safflower Oil | Soybean oil |
| Starch/Fiber | Cellulose | Cellulose | Cellulose |
| Minerals | Mineral Mix | Mineral Mix | Mineral Mix |
| Vitamins | Vitamin Mix | Vitamin Mix | Vitamin Mix |
| Micronutrient | L-Cystine | L-Cystine | L-Cystine |
| Micronutrient | Choline Bitartrate | Choline Bitartrate | Choline Bitartrate |
| Antioxidant | DL-Methionine | DL-Methionine | tBHQ |
| Other | Suspending Aid | Suspending Aid | N/A |
| Other | Moisture | Moisture | Moisture |

3B: Nutrient Food Groups

| Composition | Liquid Control (%) (F1259SP and F5736SP) | Liquid Ethanol (%) (F1258SP and F5737SP) | Chow (%) (F3197 AIN-93) |
|---|---|---|---|
| Protein | 17.0 | 17.0 | 18.1 |
| Fat | 18.0 | 18.0 | 7.1 |
| Carbohydrate | 49.0 | 23.0 | 59.3 |
| Ethanol | 0 | 26.0 | 0 |
| Fiber | 4.3 | 4.3 | 4.8 |
| Ash | 3.2 | 3.2 | 2.2 |
| Moisture | 2.1 | 2.1 | 8.5 |

3C: Liquid Diet Caloric Sources (Based on Lieber-DeCarli '82)

| Product# | F1259SP | F1258SP | F5736SP | F5737SP |
|---|---|---|---|---|
| Composition | Control (kcal/L) | Ethanol (kcal/L) | Control (kcal/L) | Ethanol (kcal/L) |
| | Casein | Casein | Soy Isolate | Soy Isolate |
| Protein | 151 | 151 | 151 | 151 |
| Fat | 359 | 359 | 359 | 359 |
| Carbohydrate | 490 | 230 | 490 | 230 |
| Ethanol | 0 | 260 | 0 | 260 |
| Total | 1000 | 1000 | 1000 | 1000 |

BioServ (Flemington, New Jersey, USA) was the source of diets. Liquid diets were prepared by reconstituting the commercial powdered meals with water and ethanol according to the manufacturer's instructions.

**Supplementary Table 4: Effects of Dietary Protein Source and Ethanol on Body and Brain Weights**

| Index | CC | EC | CS | ES |
|---|---|---|---|---|
| **Dam Body Weight-GD0** | 259.5 ± 7.5 | 257.9 ± 9.4 | 279.9 ± 16.9 | 262.6 ± 2.3 |
| **Dam Body Weight-GD21** | 283.2 ± 24.73 | 281.1 ± 21.75 | 297.9 ± 20.74 | 280.4 ± 16.72 |
| **Dam gm(%) Weight Gain** | 20.34 ± 4.02 | 20.47 ± 0.99 | 19.76 ± 4.42 | 21.41 ± 2.59 |
| **#Offspring #(%)-P0** | M: 11(42.3%)<br>F: 15 (57.7%)<br>Total: 26 | M: 12 (48.0%)<br>F:13 (52.0%)<br>Total: 25 | M: 16 (39%)<br>F:25 (61%)<br>Total: 41 | M: 17 (56.7%)<br>F: 13 (43.3%)<br>Total: 30 |
| **Offspring Birth Weight-P0** | 6.96 ± 0.39 | 7.10 ± 0.37 | 7.08 ± 0.58 | 6.88 ± 0.79 |
| **Offspring Body Weight-P35** | 130.4 ± 10.39 | 138.4 ± 14.63 | **147.1 ± 5.01** | **151.9 ± 13.82** |
| **Offspring Brain Weight-P35** | 1.75 ± 0.018 | **1.65 ± 0.02** | 1.76 ± 0.04 | 1.77 ± 0.08 |

Inter-group comparisons of the: 1) Dams' initial (Gestation Day 0-GD0) body weights (N=4/group); 2) dams' body weights at delivery (GD21) (N=4/group); 3) percentage body weight gained by the dams from GD0 to GD21; 4) offspring #(%) Male and Female delivered on P0 (Chi-square test for proportional sex differences among the groups =2.361; N.S.); 5) Offsprings' birth weights on postnatal Day 0 (P0); 6) offsprings' body weights at P35; and 7) offsprings' brain weights at P35. P35 was the experimental endpoint. The weights correspond to mean ± S.D (gms). There were 4 dams per group. Inter-group statistical comparisons of the mean values were made by Two-Way ANOVA (See Table 1). The significantly different P35 mean body and brain weights in the offspring are highlighted with bold font. Post hoc Tukey multiple comparisons test results for significant inter-group differences ($p \leq 0.05$) are highlighted with bold font. Abbreviations: CC=Control Casein; EC=Ethanol-Casein; CS=Control Soy; ES= Ethanol Soy.

**Supplementary Table S5: Analysis of Sex Effects; Mixed-Model ANOVA with the Šídák's Multiple Comparisons Test**

## A. Offsprings' Body and Brain Weights

| Parameter | CC | EC | CS | ES |
|---|---|---|---|---|
| P0 Body Weight | t=0.155; ns | t=0.122; ns | t=0.336; ns | t=0.103; ns |
| P35 Body Weight | **t=4.743;** **** | **t=6.776;**** | **t=12.5;** **** | **t=7.922;** **** |
| P35 Brain Weight | t=0.005; ns. | t=0.035; ns | t=0.021; ns | t=0.030; ns |

## B. Akt Pathway

| Parameter | CC (t; p-value) | EC (t; p-value) | CS (t; p-value) | ES (t; p-value) |
|---|---|---|---|---|
| AKT | 2.003; ns | 1.063; ns | 0.470; ns | 0.409; ns |
| GSK-3β | 1.284; ns | 1.847; ns | 1.027; ns | 1.339; ns |
| IGF1-R | 0.113; ns | 0.0012; ns | 0.653; ns | 0.883; ns |
| IN-R | 0.114; ns | 0.038; ns | 0.444; ns | 0.736; ns |
| IRS-1 | 0.345; ns | 0.094; ns | 0.007; ns | 0.055; ns |
| p-AKT | 0.014; ns | 2.413; ns | **4.249;** *** | 2.796; ns |
| p-GSK-3β | 0.081; ns | 0.168; ns | 0.307; ns | 0.1696; ns |
| p-IGF1-R | 0.089; ns | 0.115; ns | 0.168; ns | 0.1009; ns |
| p-IN-R | 0.092; ns | 0.089; ns | 0.251; ns | 0.1632; ns |
| p-IRS-1 | 0.042; ns | 0.0319; ns | 0.0187; ns | 0.0193; ns |
| pT-AKT | 0.065; ns | 0.0819; ns | 0.208; ns | 0.1183; ns |
| pT-GSK-3β | 0.005; ns | 0.0095; ns | 0.019; ns | 0.0083; ns |
| pT-IGF1-R | 1.960; ns | 0.608; ns | 0.0703; ns | 1.229; ns |
| pT-IN-R | 2.850; ns | 1.403; ns | 0.0604; ns | 0.306; ns |
| pT-IRS-1 | 0.007; ns | 0.0126; ns | 0.059; ns | 0.0049; ns |

## C. Wnt Pathway

| Parameter | CC (t; p-value) | EC (t; p-value) | CS (t; p-value) | ES (t; p-value) |
|---|---|---|---|---|
| WNT5A | 1.116; ns | 0.284; ns | 0.0346; ns | 0.2025; ns |
| WNT5B | 0.443; ns | 1.284; ns | 0.0657; ns | 0.0106; ns |
| FZD6 | 1.762; ns | 0.136; ns | 0.3634; ns | 0.239; ns |
| FZD4 | 0.124; ns | 0.419; ns | 0.0796; ns | 0.1598; ns |
| EP300 | 1.716; ns | 0.870; ns | 1.782; ns | 1.044; ns |
| AXIN2 | 1.680; ns | 0.105; ns | 0.429; ns | 0.6154; ns |
| DIXDC | **10.04;** **** | 0.376; ns | 0.3149; ns | **3.570;** ** |

## D. Notch Pathway

| Parameter | CC (t; p-value) | EC (t; p-value) | CS (t; p-value) | ES (t; p-value) |
|---|---|---|---|---|
| ASPH | 0.901; ns | 0.3075; ns | 0.3873; ns | 1.461; ns |
| NOTCH1 | 1.109; ns | 0.5184; ns | **4.668; ***** | 1.188; ns |
| JAGGED1 | 0.775; ns | 0.3330; ns | 0.0550; ns | 0.2031; ns |
| HES1 | 0.0403; ns | 0.0055; ns | 0.0195; ns | 0.0157; ns |
| HIF-1$\alpha$ | **3.852; **** | 1.503; ns | 0.2761; ns | 0.4749; ns |
| FIH | 2.216; ns | 1.371; ns | 0.1280; ns | 0.5830; ns |

Two-way Mixed Model ANOVA with the Šídák's Multiple Comparisons Test were used to compare males to females in each subgroup. The tabulated results display the t-statistics followed by significance level: ns= not significant; **p<0.0021; ***p<0.0002; ****p<0.0001. DF for STable A = 118. DF for STables B-D=36 (4 male and 4 female samples were included in each CC, EC, CS, and ES group). Abbreviations: CC=Control Casein; EC=Ethanol-Casein; CS=Control Soy; ES= Ethanol Soy. Male-Female body weight differences were significant at the experimental endpoint (P35) but not on P0, and there were no sex differences with respect to brain weight. Only 5 of the 112 (4.46%) comparisons of immunoreactivity or mRNA expression were significant (highlighted with bold font).
